# Multiple Strategies for the Application of Medicinal Plant-Derived Bioactive Compounds in Controlling Microbial Biofilm and Virulence Properties

**DOI:** 10.3390/antibiotics14060555

**Published:** 2025-05-29

**Authors:** Mulugeta Mulat, Riza Jane S. Banicod, Nazia Tabassum, Aqib Javaid, Abirami Karthikeyan, Geum-Jae Jeong, Young-Mog Kim, Won-Kyo Jung, Fazlurrahman Khan

**Affiliations:** 1 Department of Biotechnology, School of Bioscience and Technology, College of Natural Sciences, Wollo University, Dessie 1145, Ethiopia; mulugeta.mulat@wu.edu.et; 2 Fisheries Postharvest Research and Development Division, National Fisheries Research and Development Institute, Quezon City 1128, Philippines; riza.banicod@nfrdi.da.gov.ph; 3 Marine Integrated Biomedical Technology Center, The National Key Research Institutes in Universities, Pukyong National University, Busan 48513, Republic of Korea; nazia99@pukyong.ac.kr (N.T.); jgj1994@pukyong.ac.kr (G.-J.J.); ymkim@pknu.ac.kr (Y.-M.K.); wkjung@pknu.ac.kr (W.-K.J.); 4 Research Center for Marine Integrated Bionics Technology, Pukyong National University, Busan 48513, Republic of Korea; 5 Interdisciplinary Program of Marine and Fisheries Sciences and Convergent Technology, Pukyong National University, Busan 48513, Republic of Korea; aqibj@pukyong.ac.kr; 6 Industry 4.0 Convergence Bionics Engineering, Pukyong National University, Busan 48513, Republic of Korea; abirami@pukyong.ac.kr; 7 Department of Food Science and Technology, Pukyong National University, Busan 48513, Republic of Korea; 8 Major of Biomedical Engineering, Division of Smart Healthcare, College of Information Technology and Convergence and New-Senior Healthcare Innovation Center (BK21 Plus), Pukyong National University, Busan 48513, Republic of Korea; 9 Ocean and Fisheries Development International Cooperation Institute, Pukyong National University, Busan 48513, Republic of Korea; 10 International Graduate Program of Fisheries Science, Pukyong National University, Busan 48513, Republic of Korea

**Keywords:** antimicrobial resistance, medicinal plant secondary metabolites, biofilm inhibition, virulence attenuation, nanoformulations for biofilm control, drug combination

## Abstract

Biofilms are complex microbial communities encased within a self-produced extracellular matrix, which plays a critical role in chronic infections and antimicrobial resistance. These enhance pathogen survival and virulence by protecting against host immune defenses and conventional antimicrobial treatments, posing substantial challenges in clinical contexts such as device-associated infections and chronic wounds. Secondary metabolites derived from medicinal plants, such as alkaloids, tannins, flavonoids, phenolic acids, and essential oils, have gained attention as promising agents against biofilm formation, microbial virulence, and antibiotic resistance. These natural compounds not only limit microbial growth and biofilm development but also disrupt communication between bacteria, known as quorum sensing, which reduces their ability to cause disease. Through progress in nanotechnology, various nanocarriers such as lipid-based systems, polymeric nanoparticles, and metal nanoparticles have been developed to improve the solubility, stability, and cellular uptake of phytochemicals. In addition, the synergistic use of plant-based metabolites with conventional antibiotics or antifungal drugs has shown promise in tackling drug-resistant microorganisms and revitalizing existing drugs. This review comprehensively discusses the efficacy of pure secondary metabolites from medicinal plants, both as individuals and in nanoformulated forms or in combination with antimicrobial agents, as alternative strategies to control biofilm-forming pathogens. The molecular mechanisms underlying their antibiofilm and antivirulence activities are discussed in detail. Lastly, the current pitfalls, limitations, and emerging directions in translating these natural compounds into clinical applications are critically evaluated.

## 1. Introduction

Antimicrobial resistance (AMR) has emerged as a critical global health threat, recognized by the World Health Organization as one of the top ten public health challenges facing humanity [1]. The widespread misuse and overuse of antibiotics in both human and veterinary medicine have accelerated the emergence and dissemination of resistant microbial strains worldwide, with profound health, economic, and societal consequences [2,3]. Traditional antimicrobial therapies are often ineffective against biofilm-associated infections, highlighting the urgent need for innovative strategies that target biofilm formation, persistence, and virulence [4,5].

Biofilms are structured, multicellular communities of microorganisms encased in a self-produced extracellular matrix, referred to as the matrixome, composed of polysaccharides, proteins, nucleic acids, and lipids [6,7]. This matrix protects embedded cells from host immune responses and antimicrobial agents, thus contributing to chronic and recurrent infections [7,8]. Biofilms exhibit increased antimicrobial tolerance due to limited drug penetration, metabolic heterogeneity, stress adaptation, and enhanced mutation rates [5,9]. Furthermore, they support intercellular communication through quorum sensing (QS) and facilitate horizontal gene transfer (HGT), which further promotes microbial survival, virulence, and resistance [10,11]. These complex microbial communities can form on abiotic surfaces such as medical devices, as well as within host tissues, where they drive persistent infections and complicate therapeutic management [4,5,11,12,13,14].

Medicinal plants have gained increasing attention as promising sources of bioactive compounds with antimicrobial, antibiofilm, and antivirulence activities. Plant-derived secondary metabolites, such as terpenes, flavonoids, alkaloids, and phenolic compounds, have demonstrated the ability to inhibit microbial growth, disrupt biofilm formation, and interfere with virulence factor production and QS pathways [15,16]. Unlike conventional antibiotics, these phytochemicals exhibit diverse mechanisms of action and may reduce the risk of resistance development [17,18]. Some of these compounds also function as antibiotic adjuvants, efflux pump inhibitors, and QS modulators [19,20].

To enhance the therapeutic efficacy of these bioactive compounds, nanotechnology-based delivery systems have been increasingly explored. Nanocarriers, including lipid-based systems, polymeric nanoparticles, and metallic nanoparticles, can improve the solubility, stability, and cellular uptake of phytochemicals, thus boosting their antimicrobial and antibiofilm performance. Green synthesis approaches have also gained interest to formulate eco-friendly and biocompatible nanomedicines [21]. Moreover, combination therapies that pair plant-derived compounds with conventional antibiotics or antifungals have shown synergistic effects against multidrug-resistant (MDR) pathogens, potentially revitalizing existing antimicrobials [22].

Despite their promise, the clinical application of plant-based compounds faces challenges such as poor aqueous solubility, low bioavailability, and rapid metabolism [23]. Nonetheless, integrating these compounds into advanced drug delivery systems and synergistic treatment strategies offers a promising pathway toward next-generation therapeutics for biofilm-related infections [24,25].

This review provides a comprehensive overview of multiple strategies for utilizing medicinal plant-derived bioactive compounds to control microbial biofilm formation and virulence. It covers (i) the use of pure secondary metabolites and crude plant extracts, (ii) the development and advantages of nanoformulations, (iii) the synergistic effects of combining phytochemicals with conventional antimicrobial agents, and (iv) the current limitations and future research directions to overcome challenges in clinical translation. Understanding and advancing these approaches holds transformative potential to harness plant-derived compounds and redefine strategies for controlling biofilm-associated infections and microbial pathogenicity.

## 2. Mechanisms of Biofilm Formation and Virulence in Microbial Pathogens

**Figure 1:**
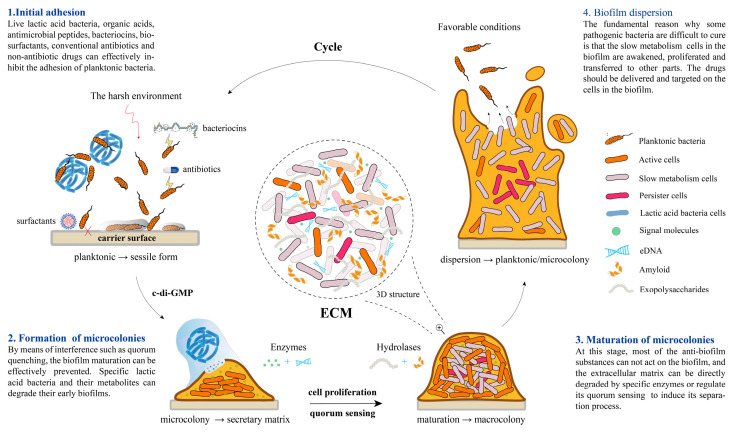
Stages of microbial biofilm formation and the composition of the biofilm matrix. Reprinted from [33], Copyright © 2024 by the authors. Licensee MDPI, Basel, Switzerland under the Creative Commons Attribution (CC BY) license (https://creativecommons.org/licenses/by/4.0/).

Microbial pathogens have evolved sophisticated survival strategies, among which biofilm formation is a central mechanism contributing to chronic infections and antimicrobial resistance. Biofilm development occurs in various stages, beginning with reversible attachment, followed by irreversible adhesion, EPS production, maturation, and eventual dispersal (Figure 1) [26,27]. The EPS matrix, composed primarily of polysaccharides, proteins, lipids, and extracellular DNA, provides structural integrity, facilitates adhesion, and protects embedded cells from antibiotics, host immune responses, and environmental stressors [7,10,28,29]. This matrix also enhances HGT, enabling rapid adaptation and resistance gene dissemination within the microbial community. In addition, QS, a bacterial communication system based on population density, regulates biofilm formation and virulence gene expression [30,31]. Through QS, bacteria coordinate the production of toxins, adhesins, and enzymes necessary for colonization, immune evasion, and persistence. The failure of conventional antibiotics to eradicate biofilm-related infections underscores the urgent need for alternative therapies targeting QS systems and biofilm integrity [30,32].

### 2.1. Structural Complexity and Resistance Mechanisms

The complex structure of biofilms, characterized by EPS and cellular heterogeneity, underpins the resilience of microbial communities against host immune defenses and antimicrobial therapies [34,35]. The EPS matrix not only provides mechanical stability but also functions as a physicochemical barrier that impedes the diffusion and efficacy of antimicrobial agents [7,36]. The composition and protective capacity of EPS vary across species, with certain matrix components providing distinct defense mechanisms. In *Pseudomonas aeruginosa*, the exopolysaccharide Psl plays a key role by sequestering cationic antibiotics such as tobramycin through ionic interactions, thereby restricting drug penetration into deeper biofilm layers [37,38]. Similarly, in *Candida albicans*, β-glucan produced by the Fks1p enzyme binds and neutralizes antifungal agents, contributing to persistent infections by lowering drug bioavailability [39].

Biofilms also contain phenotypically diverse subpopulations, notably metabolically quiescent persister cells. These cells do not carry resistance-conferring mutations but survive antimicrobial treatment by adopting a dormant physiological state, rendering them tolerant to most antibiotics [40,41]. This phenotypic heterogeneity is driven by nutrient depletion, oxygen gradients, and stress responses within biofilm microenvironments, creating zones of differential metabolic activity that enhance overall community survival [42,43].

In addition, biofilms serve as hotspots for HGT, including conjugation, transformation, and transduction, facilitating the spread of antibiotic resistance genes among cohabiting microorganisms [44,45,46]. The high cell density and close physical proximity within the matrix accelerate this genetic exchange. More importantly, biofilm-associated resistance is often mediated not by permanent genetic changes but by the regulated expression of wild-type genes in response to environmental signals and QS pathways [47].

### 2.2. Virulence Factor Expression and Quorum Sensing

Microbial biofilms elevate pathogenicity through the coordinated expression of virulence factors and the protective structure of the biofilm matrix. Key virulence determinants include toxins, proteolytic enzymes, adhesins, and immune-modulating molecules, which collectively facilitate colonization, host invasion, and immune evasion [48,49,50]. The regulation of these factors is tightly controlled by QS, a cell-density-dependent communication system that relies on the production and detection of signaling molecules such as autoinducing peptides (AIPs) in Gram-positive bacteria and N-acyl homoserine lactones (AHLs) in Gram-negative species [51,52]. QS not only governs virulence gene expression but also orchestrates biofilm development, maturation, and dispersal, while simultaneously modulating resistance-associated pathways [53,54]. This makes QS a central node in the pathophysiology of biofilm-associated infections.

**Figure 2:**
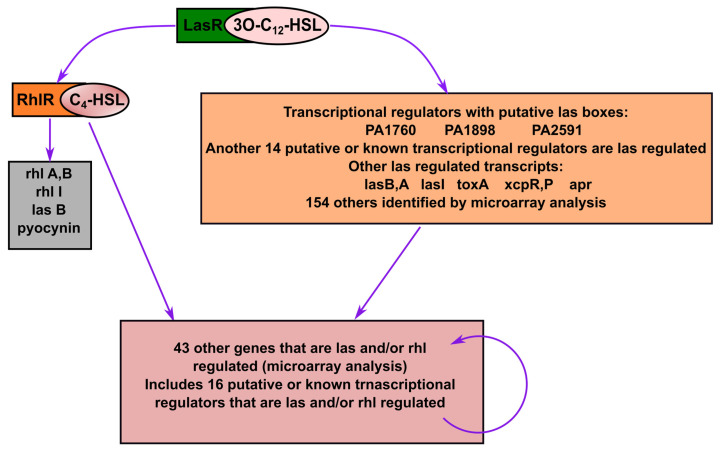
Transcriptomic analysis revealed a complex quorum sensing (QS) system in *P. aeruginosa*, involving the two well-studied QS systems (*las* and *rhl*), which are crucial for producing virulence factors, antibiotic resistance, and biofilm development. Modified figure from [56] with Copyright © 2004 Elsevier Ltd.

Given its pivotal role, quorum quenching or the disruption of QS signaling, has emerged as a promising antivirulence strategy that circumvents the selective pressure imposed by conventional antibiotics [30,55]. The mechanisms include the enzymatic degradation of QS signals, inhibition of signal synthesis, and antagonism of QS receptors. In *P. aeruginosa*, a model organism for QS studies, the *las* and *rhl* systems tightly regulate both biofilm formation and virulence expression (Figure 2) [56,57,58,59]. A notable example of plant-derived quorum quenching is andrographolide, a diterpenoid lactone from *Andrographis paniculata*, which downregulates *lasR* gene expression. This disruption attenuates QS-regulated phenotypes, including biofilm formation, protease activity, and swarming motility in *P. aeruginosa*, demonstrating the potential of phytochemicals as QS inhibitors [60].

## 3. Plant Metabolites as Natural Antibiofilm and Virulence Agents

**Figure 3:**
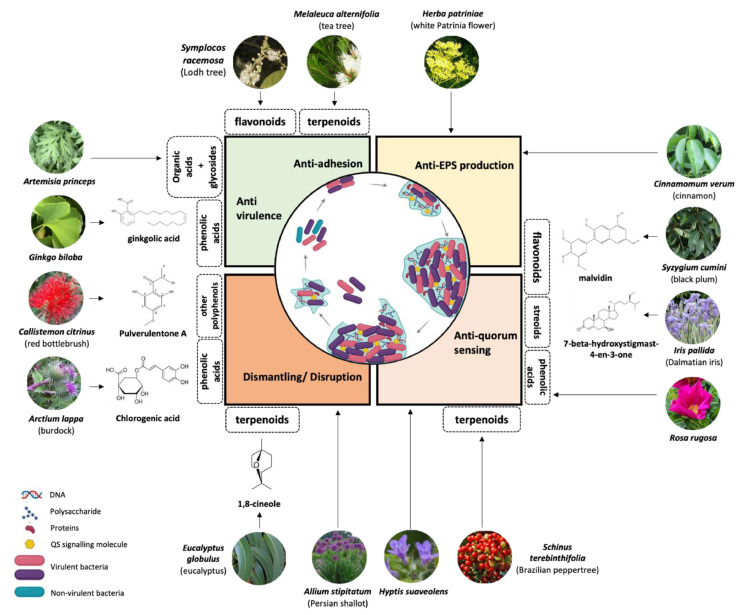
Multiple action mechanisms in the inhibitory role of medicinal plant products towards the biofilm (inhibition at the initial stage and eradication of mature biofilm), QS signaling systems, virulence factors, and EPS production. Reprinted with permission [65], Copyright © 2023 by the authors. Published by Elsevier GmbH.

The bioactive secondary metabolites found in medicinal plants exhibit potent antibacterial and antibiofilm properties. Alkaloids, tannins, terpenes, and flavonoids are among the key compounds that inhibit microbial growth and biofilm formation through diverse mechanisms [61,62,63]. In addition, essential oils (EOs) from medicinal plants have shown promising antibiofilm activity against various pathogens [64,65]. These metabolites can alter biofilm structure and disrupt QS pathways, underscoring their potential as natural alternatives to conventional antimicrobial agents. The specific activities and molecular mechanisms of plant-derived metabolites in preventing biofilm formation and reducing microbial pathogenicity are elucidated in Figure 3.

Table 1 provides an overview of plant-derived compounds with strong antibiofilm and anti-QS activities, detailing their chemical structures, plant sources, target microorganisms, effective concentrations, and specific mechanisms of action. The listed compounds demonstrate the ability to interfere with QS signaling and biofilm development in major pathogens such as *P. aeruginosa*, *Staphylococcus aureus*, and *Escherichia coli*, among others.

### 3.1. Alkaloids

Alkaloids derived from medicinal plants exhibit strong antivirulence and antibiofilm activities through diverse mechanisms. Notably, compounds such as piperine and berberine inhibit biofilm formation by disrupting QS pathways, compromising biofilm structural integrity via the suppression of EPS production, and reducing microbial virulence by downregulating genes involved in toxin synthesis and bacterial motility [66,67,68,69,70]. 1,3,4-oxadiazole derivatives isolated from plants have been shown to inhibit the production of *P. aeruginosa* toxin pyocyanin and its QS precursor, 2-heptyl-4-quinolone (HHQ), effectively interfering with QS-mediated pathogenicity. Similarly, 7-hydroxyindole modulates the expression of multiple virulence-associated genes and inhibits swarming motility, a critical factor for surface colonization and biofilm expansion [71,72]. Another promising phytochemical hordenine—a dietary alkaloid found in barley—functions as a QS inhibitor and synergizes with aminoglycoside antibiotics against *P. aeruginosa*. Hordenine reduces the production of acyl-homoserine lactones (AHLs), the primary signaling molecules in Gram-negative QS systems, resulting in decreased biofilm biomass, bacterial motility, and the secretion of virulence factors including elastase, protease, rhamnolipids, pyoverdine, and pyocyanin. These factors are essential for tissue damage, immune evasion, and iron acquisition, underscoring the therapeutic potential of alkaloids in combating biofilm-associated infections [73].

### 3.2. Tannins

Tannins, a class of polyphenolic compounds derived from medicinal plants, have demonstrated antibiofilm and antivirulence activities through multiple interconnected mechanisms [74]. These compounds are primarily classified into two groups, hydrolyzable tannins, which are esters of gallic acid, and condensed tannins, commonly known as proanthocyanidins (PACs), which are polymers composed of polyhydroxyflavan-3-ol units [75]. A well-studied hydrolyzable tannin, tannic acid, found in sources such as gallnuts and tea, exerts its antibiofilm effects by chelating essential metal ions within the EPS matrix. Since metal ions are critical for maintaining the biofilm structure and facilitating bacterial adhesion, their sequestration destabilizes the biofilm architecture [76,77,78]. Additionally, tannic acid targets bacterial cell wall proteins, disrupts QS pathways, and downregulates adhesion genes such as *agrA*, *icaA*, and *icaD* in *S. aureus* [79]. Proanthocyanidins extracted from plants like *Anadenanthera colubrina* and *Caesalpinia leptophloeos* have also been reported to inhibit microbial biofilm adhesion, while hydrolyzable tannins from *Myracrodruon urundeuva* display bacteriostatic and anti-adhesive effects against *P. aeruginosa* [80]. Some tannins, such as hamamelitannin, specifically inhibit QS by suppressing the RNAIII regulator, thus modulating virulence expression [81].

### 3.3. Flavonoids

Flavonoids are a diverse group of polyphenolic compounds abundantly present in fruits, vegetables, and plant-derived beverages [82]. These secondary metabolites, often responsible for the pigmentation of flowers and plant tissues, exert antimicrobial effects by targeting various steps in biofilm development and QS regulation. Quercetin and naringenin disrupt bacterial communication networks and hinder surface adherence, thereby attenuating the virulence of a broad range of pathogens [83,84,85]. Naringenin has been shown to suppress the expression of biofilm-associated genes, compromising the structural cohesion of microbial communities [86,87,88]. In addition to modulating gene expression, flavonoids degrade the EPS matrix through metal ion chelation and antioxidant activity, further destabilizing mature biofilms [29,89].

Some studies have further elucidated flavonoid-mediated biofilm inhibition. Matilla-Cuenca et al. (2020) [90] demonstrated that quercetin, myricetin, and scutellarin suppress *S. aureus* biofilm formation by targeting Bap, an amyloid surface protein critical for biofilm assembly in certain *S. aureus* and coagulase-negative staphylococci strains, without altering the gene expression profile of the Bap pathway. This highlights a non-genomic mode of action involving interference with protein assembly. Similarly, Pruteanu et al. (2020) [91] reported that flavonoids such as luteolin, morin, myricetin, and quercetin significantly impaired macrocolony biofilms of *E. coli*, *P. aeruginosa*, and *Bacillus subtilis* by disrupting the formation of amyloid curli fibers and cellulose, a critical matrix scaffold, despite limited effects on submerged biofilms. These findings underscore the ability of flavonoids to selectively destabilize biofilm structures through both gene-dependent and structural interference mechanisms, marking them as promising candidates for antivirulence-based therapeutic strategies.

### 3.4. Essential Oils

The hydrophobic nature of essential oils (EOs), which are volatile compounds derived from aromatic medicinal plants, facilitates penetration into bacterial membranes, compromising membrane integrity and causing the leakage of essential intracellular components, ultimately leading to cell death [92,93,94]. EOs also interfere with QS by modulating regulatory genes such as *luxR*, *luxS*, and *agrBDCA*, thereby regulating the expression of virulence factors and disrupting biofilm coordination [95]. They also impair bacterial motility and adhesion by downregulating genes involved in flagella and fimbriae synthesis, which are essential for initial surface attachment and biofilm expansion [96]. Furthermore, EOs are effective not only at preventing biofilm formation but also at eradicating established biofilms on various surfaces, including medical devices [97]. Importantly, many EOs exhibit low cytotoxicity toward human cells, making them attractive candidates for novel antimicrobial therapies with improved safety profiles [98].

Empirical studies have validated the efficacy of EOs from various plant sources. For example, *Melaleuca alternifolia*, *Melissa officinalis*, and *Thymus zygis* EOs significantly reduced the biofilm biomass in *S. aureus* and *E. coli* [64,99]. Tropical plant-derived EOs, such as those from *Psiadia arguta* and *Citrus hystrix*, have shown strong antibiofilm activity against *S. epidermidis*, *E. coli*, and *C. albicans* [100]. In the context of oral health, *Cymbopogon citratus* and *Lippia alba* EOs were particularly effective against *Streptococcus mutans* biofilms [101]. Moreover, EOs from *Origanum majorana* and *Rosmarinus officinalis* demonstrated potent activity against methicillin-resistant *S. aureus* [102], while *Allium sativum* and *Cinnamomum zeylanicum* EOs exhibited notable efficacy against *Candida* spp. oral biofilms [103].

### 3.5. Terpenes

Terpenes and terpenoids are hydrocarbon-based secondary metabolites constructed from repeating five-carbon isoprene units, which represent one of the most structurally diverse and biologically potent classes of natural products found in plants [104]. These molecules have shown remarkable promise in combating biofilm-associated infections due to their broad-spectrum antimicrobial and antivirulence activities. Terpenes such as carvacrol, geraniol, and thymol exhibit potent antibiofilm effects against *Candida* species by inhibiting early-stage biofilm development, disrupting mature biofilms, and attenuating virulence factors in pathogens like *C. albicans*, *P. aeruginosa*, and *A. baumannii* [15,105,106,107,108]. Notably, carvacrol and terpinen-4-ol retain antibacterial efficacy at sub-inhibitory concentrations without affecting microbial viability, thereby reducing the selective pressure for resistance development [109,110]. Their versatility across surfaces, including polystyrene, stainless steel, and biomedical implants, adds to their clinical potential [111,112].

Mechanistically, these compounds exert their effects through the disruption of QS systems and interference with gene expression involved in virulence and biofilm formation. Carvacrol, for example, downregulates QS and stress response genes such as *speB*, *srtB*, *luxS*, *covS*, *dltA*, *ciaH*, and *hasA*, impairing bacterial communication, adhesion, and survival [113]. Triterpenoids derived from *Inula* species have been shown to suppress *Chromobacterium violaceum* biofilm formation by inhibiting QS circuits [114], while phytochemicals like cassipourol and β-sitosterol block QS-regulated gene expression in *P. aeruginosa* [107]. In addition, myrtenol enhances antibiotic susceptibility in *A. baumannii* by downregulating genes critical for biofilm maintenance and resistance [108]. The antibacterial and antifungal efficacy of terpenes is strongly influenced by their physicochemical properties. Amphiphilic compounds with molecular weights ranging between 150 and 550 g/mol are particularly effective due to their ability to penetrate microbial membranes and interact with intracellular targets [115].

### 3.6. Phenolic Acids

Phenolic acids exhibit significant antivirulence and antibiofilm properties through interfering with the signaling pathway of the QS system [116]. These compounds can interfere with bacterial communication by altering the synthesis or activity of QS signaling molecules, which reduces the formation of virulence factors and biofilms. For instance, it has been demonstrated that the well-known phenolic acid, caffeic acid, which is present in coffee and other plants, inhibits QS-regulated gene expression in *P. aeruginosa* [117,118]. This results in a decrease in the production of virulence factors like pyocyanin and elastase as well as a decrease in the formation of biofilms. Similarly, ferulic acid is another phenolic acid present in high concentrations in cereal grains, which prohibits *S. aureus* from forming biofilms by inhibiting QS-controlled pathways involved in adhesion and toxin production [116,119]. Phenolic acids have substantial antioxidant and metal-chelating qualities in addition to their QS-modulating activities. These qualities further disrupt the biofilm matrix, increasing the likelihood that they will be useful agents in the fight against infections linked to biofilms. Phenolic acids are intriguing candidates for therapeutic approaches that target bacterial illnesses linked to biofilms because of these many processes.

**Table 1:** Pure compounds from medicinal plants with antibiofilm and quorum sensing properties.

Name of the Compound	Structure of Compounds	Source Plant Species	Target Microbes	Biofilm Inhibitory Concentration (µg/mL)	Mechanism of Action	References
14-Deoxy-11,12-didehydroandrographolide	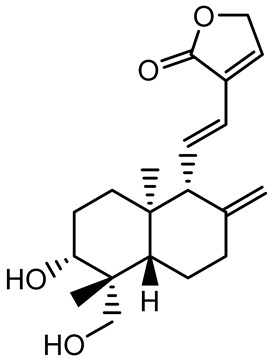	*Andrographis paniculata*	*Pseudomonas aeruginosa*	34.85	Inhibits biofilm formation by targeting the quorum sensing pathway, leading to a reduction in extracellular polymeric substances, pyocyanin production, and extracellular protease synthesis.	[120]
Andrographolide, 14-deoxyandrographolide, 14-deoxy-12-hydroxyandrographolide, Neoandrographolide	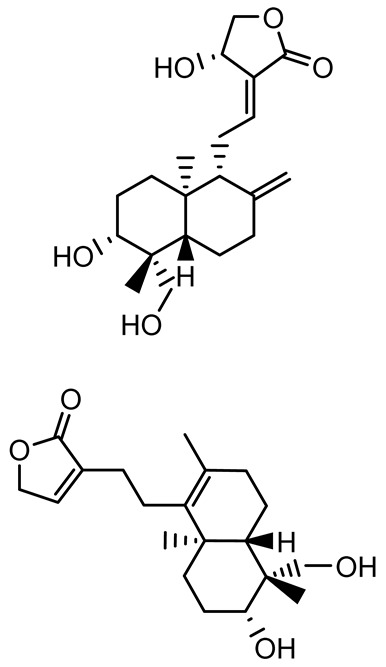 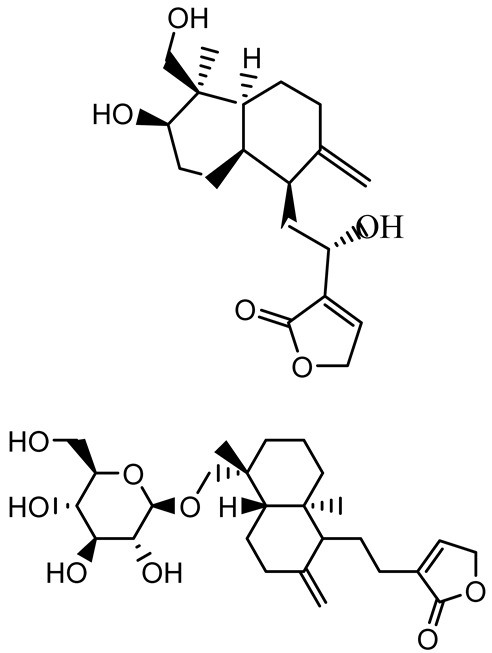		*P. aeruginosa* PA22 and PA247	310–5000	Exert their quorum quenching activity by downregulating *lasR* gene expression, thereby disrupting quorum sensing and leading to reduced biofilm formation, protease production, and swarming motility in *P. aeruginosa*.	[60]
Andrographolide	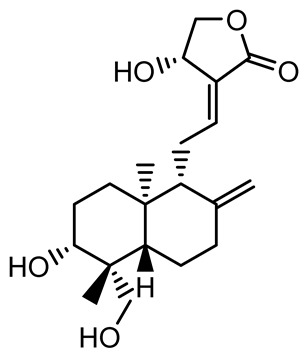		*Listeria monocytogenes* 10403S	125–1000	Inhibits the Agr QS system by downregulating *agrBDCA* genes and P2 promoter activity, leading to reduced biofilm formation, virulence gene expression, hemolytic activity, and host cell invasion.	[121]
Licochalcone A (LAA)	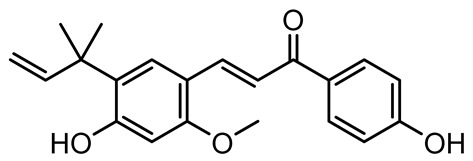	*Glycyrrhiza inflate*	*Salmonella typhimurium*	62.5	Downregulates *sdiA* gene expression, disrupting AHLs and affecting the expression of QS-controlled virulence factors, thus decreasing motility, fimbria formation, bacterial invasion, biofilm production.	[122]
Epigallocatechin-3-gallate (EGCG)	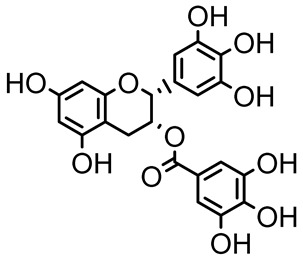	*Camellia sinensis*	*S. typhimurium*	3.125	Downregulates *luxS* gene expression, affecting QS system and some other genes involved in virulence.	
Magnolol, Honokiol	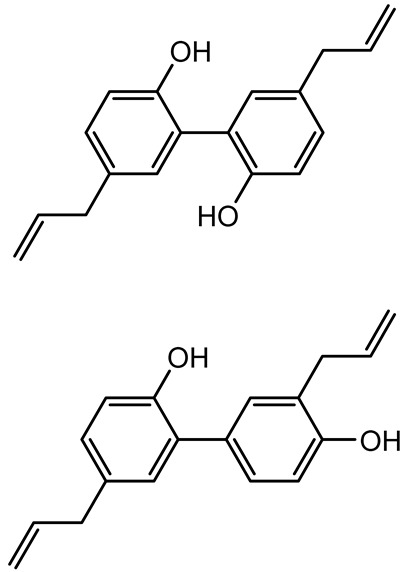	*Magnolia officinalis*	*Acinetobacter baumannii*	-	Inhibit biofilm formation, disperse mature biofilms, suppress pellicle formation and surface motilities.	[123]
Verbascoside	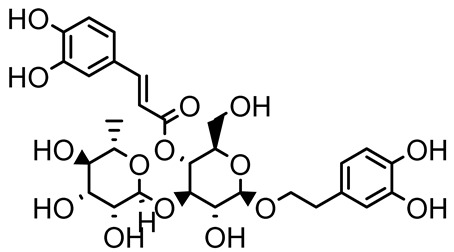	*Forsythia* spp.	*Staphylococcus aureus* USA300	≥8	Inhibits sortase A, blocking MSCRAMM anchoring to the cell wall; reduces adhesion, invasion, and biofilm formation.	[124]
Echinacoside	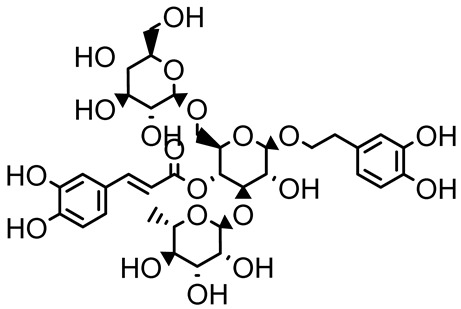	*Echinacea angustifolia*	*P. aeruginosa*	0.125–44	Inhibits diguanylate cyclase SiaD, reducing intracellular c-di-GMP levels, thereby inhibiting autoaggregation and enhancing tobramycin efficacy against biofilm aggregates.	[125]
Scutellarein	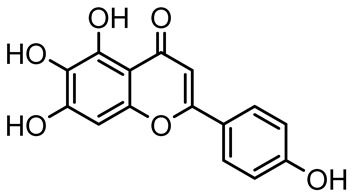	*Scutellaria baicalensis* and *Erigeron breviscapus*	*A. baumannii* ATCC 17978	32–64	Inhibits biofilm formation, motility, and bacterial persistence by targeting and inhibiting polyphosphate kinase 1 (PPK1).	[126]
Baicalin	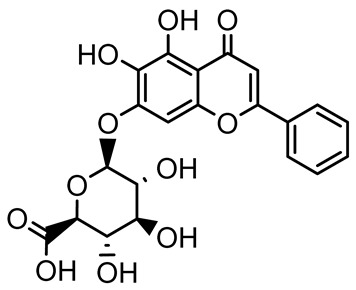	*Scutellaria baicalensis*	*P. aeruginosa*	64–256	Inhibits quorum sensing by downregulating *lasI/R*, *rhlI/R*, *pqsA/R* genes; suppresses production of QS signals (3-oxo-C12-HSL, C4-HSL); reduces biofilm, virulence factors, and motility.	[127]
			*S. saprophyticus*	31.25–250	Inhibits MsrA efflux pump; reduces ATP and pyruvate kinase activity; downregulates *agrA*, *agrC*, RNAIII, sarA; inhibits biofilm formation and quorum sensing system.	[128]
			*S. aureus* 17546	32 and 64	Inhibits quorum sensing by downregulating *agrA*, RNAIII, *sarA*, and *ica* genes; reduces virulence factors (SEA, *hla*); prevents and disrupts biofilm formation.	[129]
Wogonin	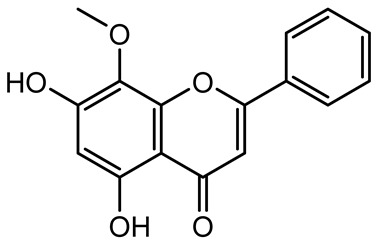	*Agrimonia pilosa*	*P. aeruginosa* PAO1	15–30	Inhibits the PQS quorum sensing system by targeting pqsA and *pqsR* genes; reduces PQS signal production; suppresses virulence factors; inhibits swimming, swarming, and twitching motility; attenuates biofilm formation and bacterial pathogenicity.	[130]
Vitexin	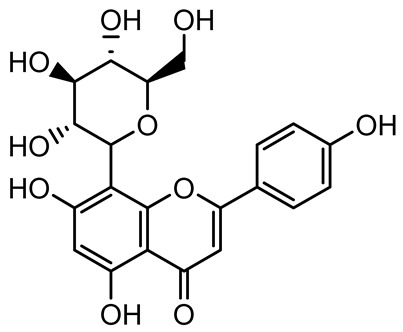	*Vitex peduncularis*	*S. aureus* (MTCC 96)	26–126	Reduces cell surface hydrophobicity, membrane depolarization, and EPS production; downregulates biofilm genes (*icaAB*, *dltA*) and QS genes (*agrAC*).	[131]
			*P. aeruginosa* (MTCC 2488)	110	Inhibits quorum sensing by targeting *LuxR*, *LasA*, *LasI*, and motility-related proteins (PilY1, PilT); reduces EPS, biofilm protein, pyocyanin, protease, LasA/B activity.	[132]
Morin	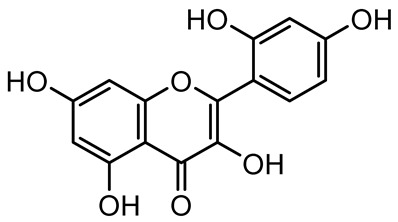	Fig, almond, guava	Methicillin-resistant *S. aureus* and Vancomycin-resistant *S. aureus*	282 398	Inhibits biofilm formation, disrupts established biofilms, reduces sliding motility, reduces EPS production, binds to SarA (global regulator), inhibiting its DNA-binding activity and thereby interfering with quorum sensing-regulated biofilm and virulence gene expression.	[133]
Naringin, Neohesperidin, Hesperidin	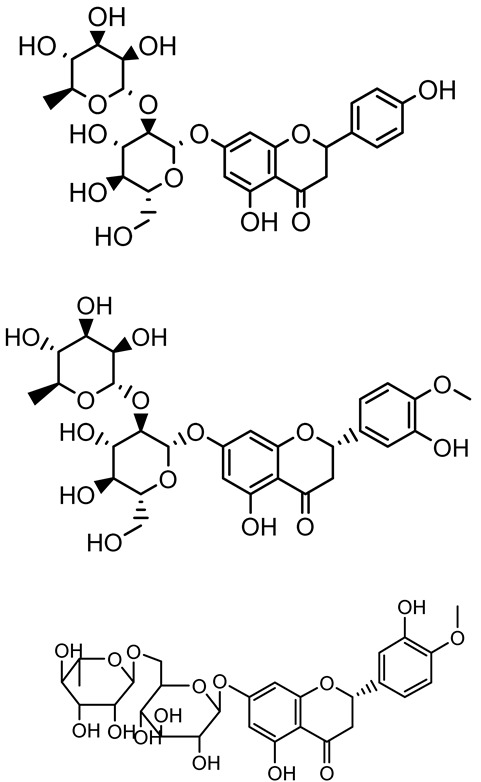	*Citrus*	*Yersinia enterocolitica*	100–400	Inhibit quorum sensing by reducing AHL (3-oxo-C6-HSL and C6-HSL) production, inhibit biofilm formation, inhibit swimming motility, alter the expression of QS-related genes (yenR, *fliA*, *flhDC*).	[134]
Diosmin, myricetin, Neohesperidin	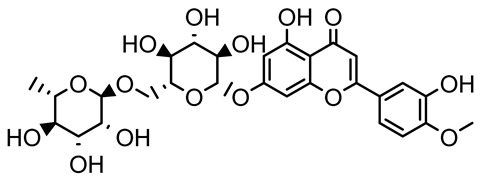 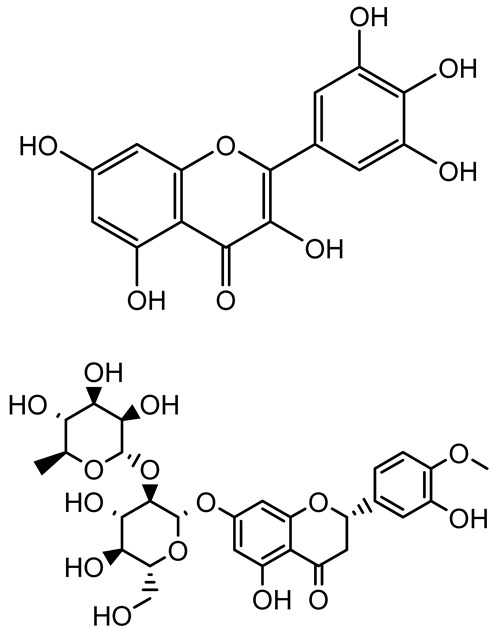	Citrus fruits	*P. aeruginosa*	50–400	Inhibit biofilm formation, reduce EPS and eDNA production, interfere with quorum sensing by downregulating genes such as *lasI*, *pvdS*, and *rhlC*.	[135]
Glabridin	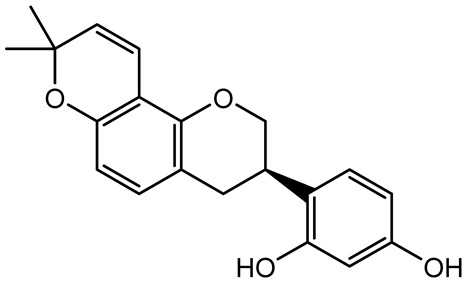	*Glycyrrhiza glabra*	*L. monocytogenes*	3.91–15.63	Reduces motility and hemolytic activity, decreases intracellular survival, inhibits hly gene expression, induces ROS in macrophages without affecting biofilm formation.	[136]
β-Glycyrrhetinic acid (BGA)	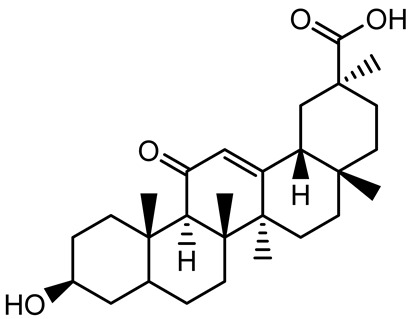	*Glycyrrhiza* spp.	*Streptococcus mutans*, *S. sobrinus*, *S. gordonii*, *Porphyromonas gingivalis*	128–256	Inhibits bacterial growth, biofilm formation, and bacterial coaggregation; suppresses plaque biofilm maturation by affecting early colonizers and preventing *P. gingivalis* adhesion.	[137]
Glycyrrhizin	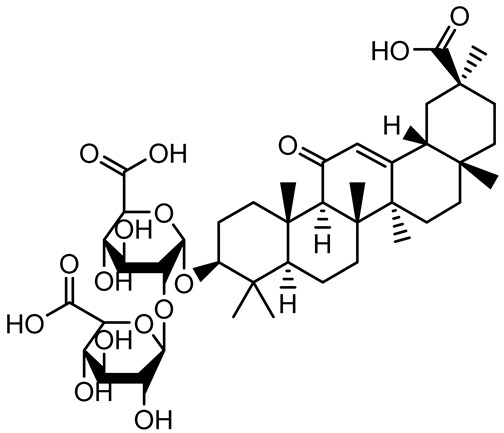	*Glycyrrhiza uralensis*	*P. plecoglossicida*	60–100	Inhibits biofilm formation, increases bacterial membrane permeability, suppresses bacterial growth, enhances phagocytosis and bactericidal capacity of host immune cells.	[138]
Carnosic acid	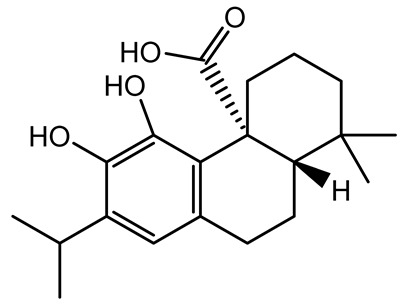	*Salvia rosmarinus*	*S. aureus*	50	Inhibits quorum sensing by downregulating *agrA* and rnaIII genes, reduces virulence genes (*hla*, *psm*α), prevents biofilm formation, enhances intracellular killing by macrophages without bactericidal effects, directly binds AgrA DNA-binding site.	[139]
Ursolic acid	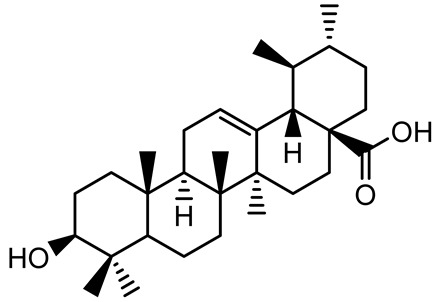	*Rosmarinus officinalis*	*S. aureus*	39	Disrupts bacterial cell wall and membrane integrity, inhibits protein synthesis, reduces biofilm formation, induces intracellular ROS production, leading to bacterial death.	[140]
Betulinic acid	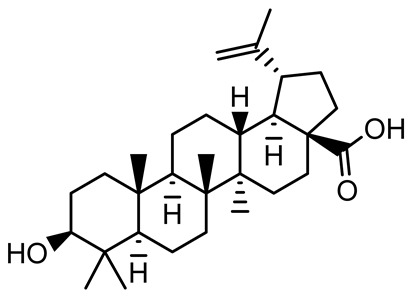	*Ludwigia grandiflora*	*S. aureus*, *Candida albicans*	25	Disrupts cell membranes and surface hydrophobicity, reduces biofilm biomass and early adhesion, downregulates *sasF* (*S. aureus*) gene expression, inhibits yeast-to-hyphae transition in *C. albicans* without microbicidal effects.	[141]
Betulin, Betulinic acid	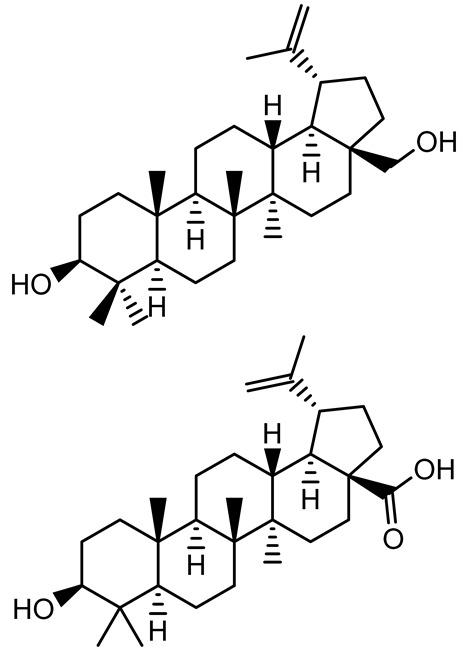	*Betula species* (birch trees), *Trochodendron aralioides*, *Ziziphus vulgaris var. spinosus*	*P. aeruginosa* PAO1	125	Inhibit quorum sensing by competitive binding to LasR and RhlR receptors; suppress virulence factors (pyocyanin, elastase, protease, rhamnolipid, chitinase); inhibit biofilm formation; reduce EPS, alginate, eDNA production, and surface hydrophobicity; impair motility.	[142]
3,5-di-O-galloylquinic acid, myricetin	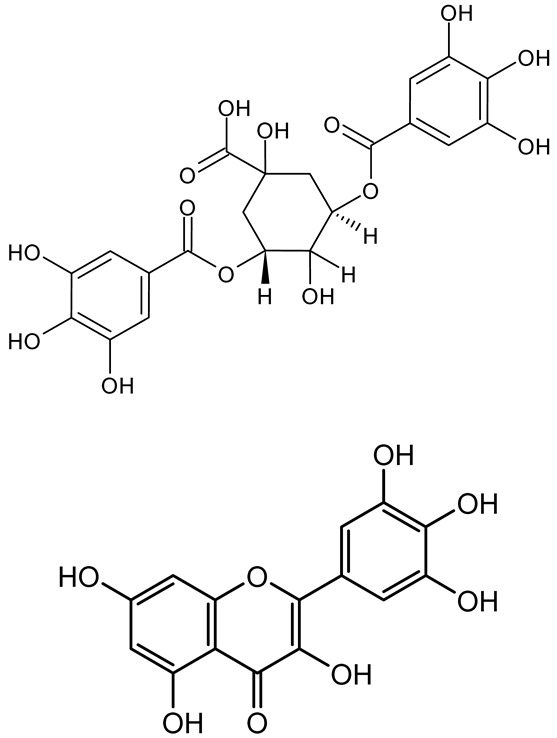	*Myrtus communis*	*Chromobacterium violaceum* 6267, *P. aeruginosa* PAO1	31.25–125	Inhibit quorum sensing by binding to QS receptor CviR; downregulate *lasI*, *lasR*, *rhlI*, *rhlR*, *pqsA* genes; inhibit biofilm formation, pyocyanin production, swarming motility, and protease activity.	[143]
Methyl gallate, Pyrogallol, Betulin, Epicatechin gallate, Dehydroabietic acid	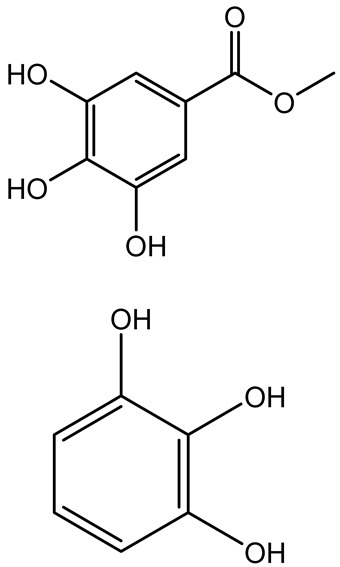 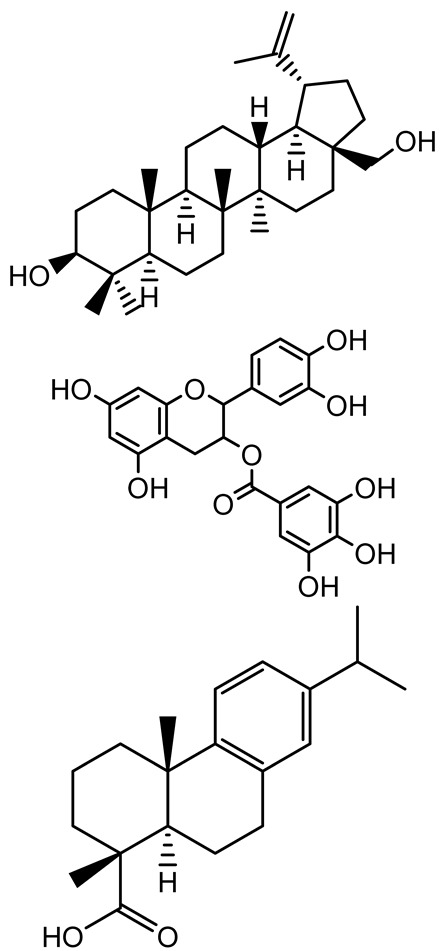	*Acacia nilotica pods*	*C. violaceum*, *P. aeruginosa*, *Serratia marcescens*	250–500	Inhibit quorum sensing by binding QS receptors (*LasI*, *LasR*, *RhlR*, *CviR*) and biofilm proteins (PilY1, PilT); suppress violacein, pyocyanin, protease, swarming, and biofilm formation; promote oxidative stress that disrupts bacterial communication and biofilm stability.	[144]
Taxifolin, quercetin, Silybin, Silychristin	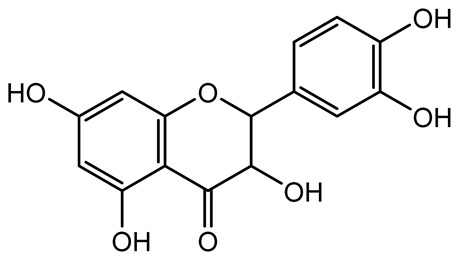 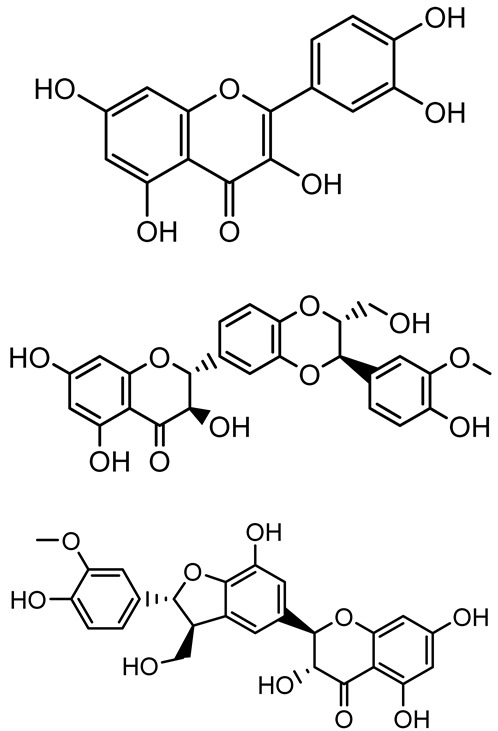	*Silybum marianum*	*Vibrio campbellii*, *S. aureus*, *P. aeruginosa*	<10 µM	Inhibit AI-1 and AI-2 quorum sensing by interfering with bacterial communication signals, prevent early bacterial surface colonization (biofilm initiation), enhance antibiotic sensitization in resistant strains.	[145]
Tormentic acid, 23-Hydroxycorosolic acid	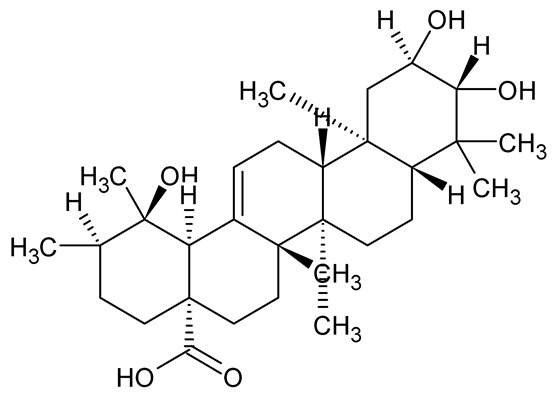 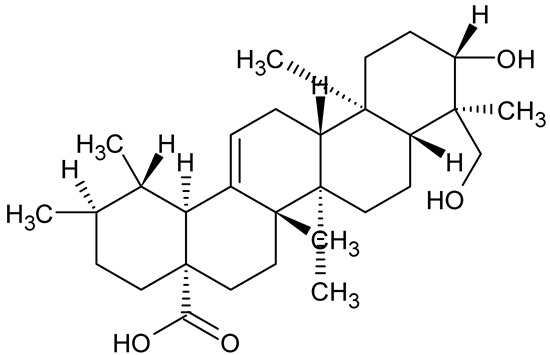	*Sarcochlamys pulcherrima*	*S. aureus*	20 25	Depolarize bacterial membrane, inhibit biofilm formation, reduce exopolysaccharide production, suppress motility and protease activity, downregulate virulence gene expression, bind strongly to biofilm and quorum sensing proteins (TarO for TA, AgrA for HCA).	[146]
Ellagic acid, gallic acid, Methyl gallate, Chlorogenic acid, naringenin, Apigenin	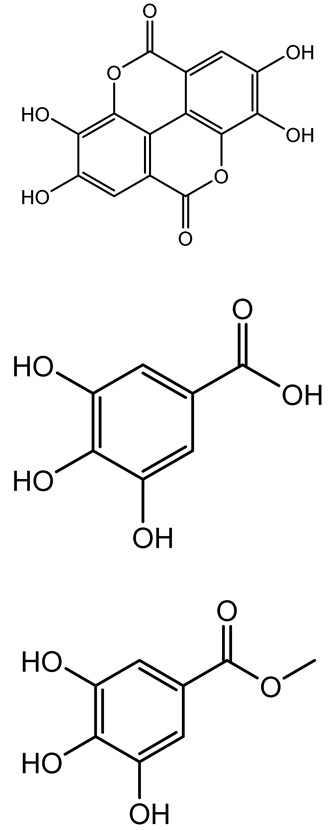 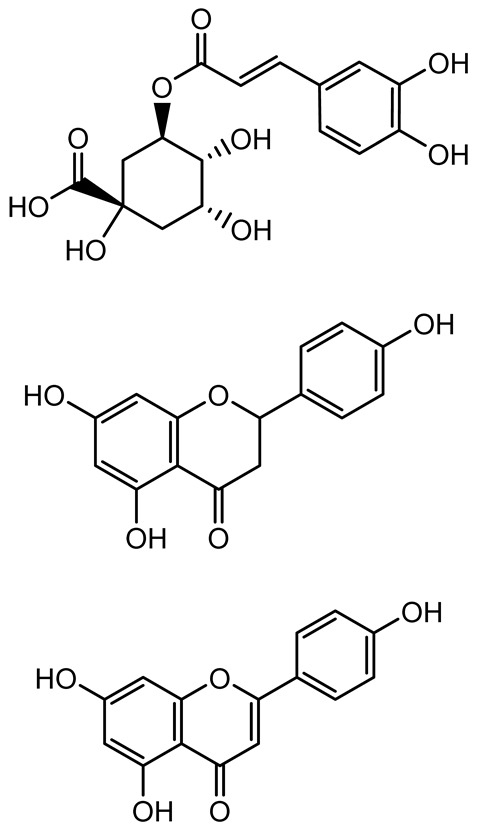	*Dioon spinulosum*	*P. aeruginosa*, *C. violaceum*	250–1000	Inhibits quorum sensing by downregulating *lasI*, *lasR*, *rhlI*, *rhlR* genes; reduces EPS production, cell surface hydrophobicity, violacein production.	[147]
Alpha-copaene, Caryophyllene, Nerolidol	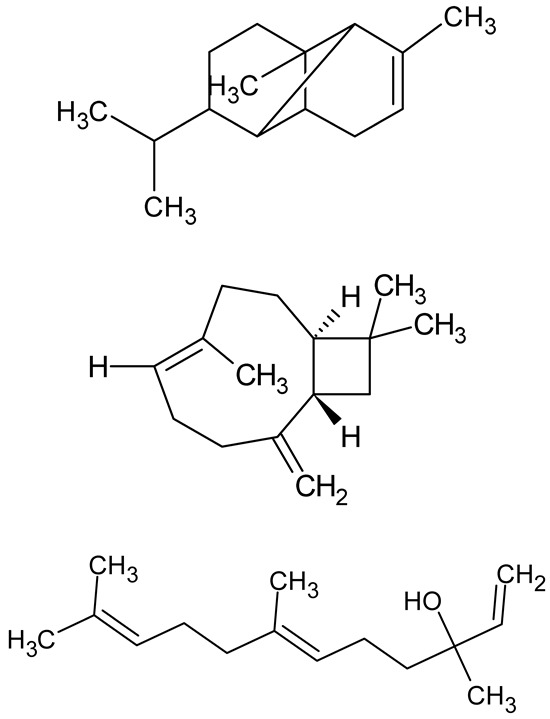	*Psidium guajava*	*C. violaceum* 12742, *P. aeruginosa* PAO1	500–1000	Inhibit quorum sensing by binding to QS receptors (*RhlR*, *CviR’*, *LasI*, *LasR*), suppress AHL production, reduce virulence factors (pyoverdin, pyocyanin, rhamnolipid), inhibit biofilm formation in a concentration-dependent manner.	[148]
Curcumin	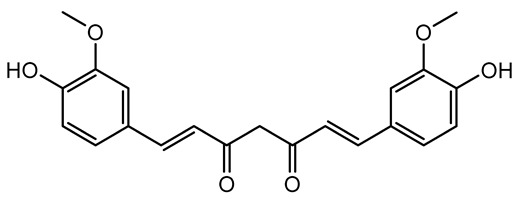	*Curcuma longa*	*Escherichia coli*, *P. aeruginosa* PAO1, *Proteus mirabilis*, *Serratia marcescens*	0.125–600	Inhibits biofilm formation and disrupts mature biofilms by interfering with quorum sensing systems, reduces EPS and alginate production, and suppresses swimming and swarming motility.	[149]
Oleanolic aldehyde coumarate	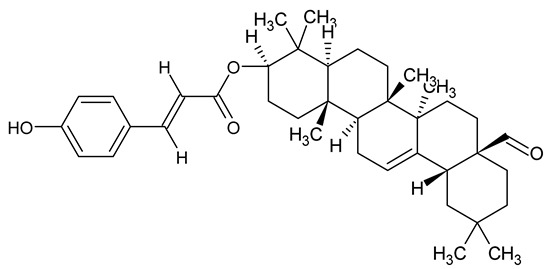	*Dalbergia trichocarpa*	*P. aeruginosa* PAO1	117	Inhibits quorum sensing systems (*las* and *rhl*), reduces AHL production, inhibits QS-regulated virulence factors, inhibits biofilm formation and maintenance, reduces extracellular polysaccharides, enhances antibiotic (tobramycin) activity against biofilm-encapsulated bacteria.	[150]
Apigenin, Acacetin, Genistein, Biochanin A, Daidzein	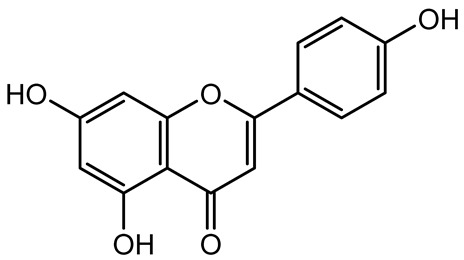 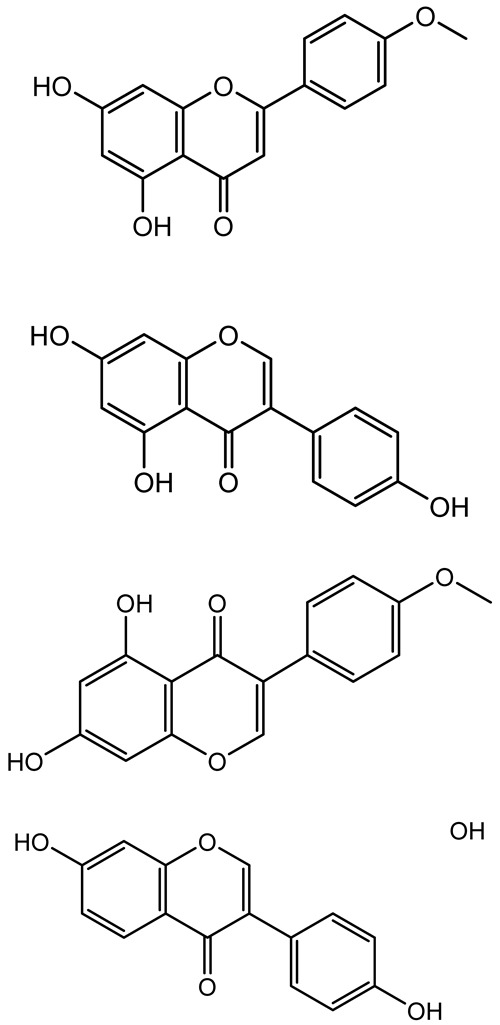	Legumes	*P. aeruginosa* PAO1	0.4875–45	Inhibit quorum sensing by downregulating *lasI*, *lasR*, *rhlI*, *rhlR* pathways; suppress virulence factors (biofilm, pyocyanin, pyoverdin, rhamnolipid, alginate, protease, exopolysaccharide); inhibit swimming and swarming motility.	[151]
Isoliquiritin, EGCG, Eugenol, Luteolin, Chrysin	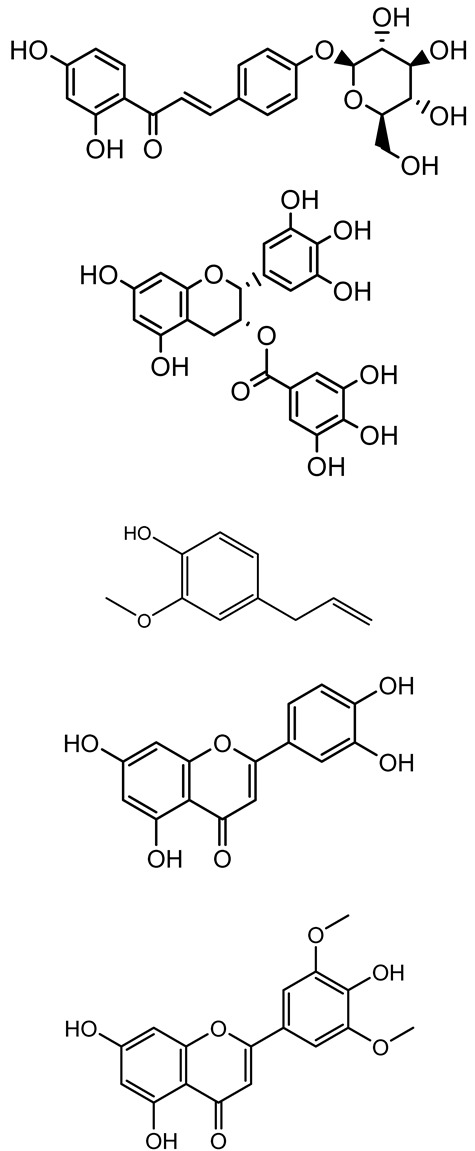	Various plants	*P. aeruginosa*	95–500	Inhibit quorum sensing by targeting *LasI*, *LasR*, *RhlI*, and *RhlR* systems; inhibit biofilm formation; reduce exopolysaccharide production, aggregation, and hydrophobicity.	[152]

## 4. Medicinal Plant Compound-Derived Nanoparticles with Antibiofilm and Virulence Properties

Nanoparticles have emerged as a powerful tool in the fight against biofilm-associated infections and the growing threat of antimicrobial resistance [153,154]. Their nanoscale dimensions and high surface area-to-volume ratio enable efficient penetration into biofilms and improved interactions with microbial cells and extracellular matrix components [155,156]. Various types of nanoparticles, such as metal-based, lipid-based, and polymeric nanoparticles, have demonstrated antibiofilm activity through mechanisms like disrupting the biofilm structure, interfering with bacterial metabolism, and delivering antimicrobial agents more effectively [157,158]. Nanoparticles can also be used to functionalize surfaces, thereby preventing biofilm formation on medical devices [159]. The interaction between nanoparticles and biofilm components, including polysaccharides, proteins, and nucleic acids, plays a crucial role in their antimicrobial efficacy [156]. As such, nanotechnology provides innovative strategies to overcome the limitations of conventional therapeutics in managing chronic and resistant infections [160].

Recent advances have focused on the green synthesis of nanoparticles using medicinal plant-derived compounds, which offer biocompatibility and additional therapeutic value. For instance, silver, gold, and zinc oxide nanoparticles synthesized from *Crataeva nurvala* bark extract and phloroglucinol, respectively, have been shown to effectively inhibit both biofilm formation and virulence traits in *P. aeruginosa* [161,162]. Tetramethylpyrazine-coated gold nanoparticles have also been reported to reduce biofilm biomass and suppress virulence factors [163]. Other studies have reported the effectiveness of silver nanoparticles synthesized from various medicinal plants against multiple bacterial pathogens [164]. In another study, eugenol-functionalized magnetite nanoparticles effectively modulated virulence expression and persistence in clinical strains of *P. aeruginosa* [165].

The mechanisms underlying the antimicrobial activity of plant-derived nanoparticles are multifaceted, including the disruption of bacterial membranes, generation of reactive oxygen species (ROS), and downregulation of genes associated with QS, biofilm maturation, and virulence expression [166,167]. These unique features not only enhance their antimicrobial efficacy but also reduce the likelihood of resistance development compared to traditional antibiotics [153,168]. These findings underscore the immense potential of medicinal plant compound-derived nanoparticles as next-generation therapeutics for managing biofilm-related infections. The following sections explore specific nanomaterial formulations and their applications in regulating biofilm development and virulence across bacterial and fungal pathogens.

### 4.1. Nanomaterials for Effective Delivery of Plant Compounds

Compounds derived from medicinal plants usually encounter challenges in their application, such as low bioavailability, rapid degradation, and poor solubility [169]. Nanotechnology has shown great promise in overcoming these limits by developing enhanced delivery systems that improve the stability, solubility, and selective distribution of bioactive chemicals. To improve the therapeutic efficacy of plant-derived compounds, nanocarriers such as metal nanoparticles, polymeric nanoparticles, and lipid-based systems have been extensively studied [170,171,172]. These innovative delivery systems enhance the compounds’ antibacterial and antivirulence properties by preventing degradation and allowing their efficient transfer to the intended site of action. These nanocarriers enable targeted, regulated release, thereby enhancing the therapeutic efficacy of plant-derived bioactive chemicals.

#### 4.1.1. Lipid-Based Nanocarriers

**Figure 4:**
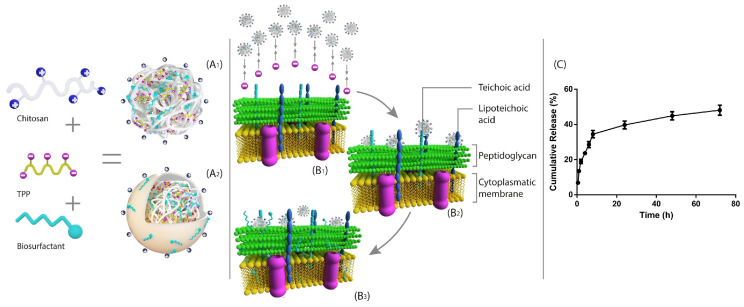
Antimicrobial activity of RLs-CSp towards *S. aureus*. RLs can be adsorbed to the surface of RLs-CSp (**A_2_**) when encapsulated onto RLs-CSp (**A_1_**), which is another option. Through electrostatic attraction (**B_1_**,**B_2_**), first, the negatively charged *S. aureus* membranes are drawn to the positively charged RLs-CSp. RLs are discharged from the RLs-CSp delivery system and penetrate membranes over time, ultimately killing and destroying these cells (**B_3_**). The experimental in vitro RLs’ cumulative release profile (**C**) shows that all chemicals can be released from the RLs-CSp. Reprinted with permission [181], Copyright © 2020 Elsevier Ltd.

Lipid-based nanocarriers, including solid lipid nanoparticles (SLNs), liposomes, and nanoemulsions, are sophisticated drug delivery methods frequently employed to encapsulate hydrophobic substances [173,174,175]. These lipid carriers help to increase the bioavailability of the encapsulated drugs and shield them from enzymatic breakdown. Liposomes provide greater biocompatibility by mimicking natural cellular membranes through the use of lipid bilayers [176,177]. At physiological temperatures, solid lipid nanoparticles remain solid, providing stability and regulated release characteristics. Drugs that are hydrophobic can be made more soluble with the help of nanoemulsions [178,179,180]. These lipid-based nanocarriers work together to efficiently transport bioactive chemicals across biological membranes, ensuring customized delivery to specific organs or tissues. This modified distribution is critical for minimizing side effects while increasing the effectiveness of treatments. Recent studies have highlighted the potential of rhamnolipids (RLs) and other antimicrobial compounds in combating *S. aureus* infections, including drug-resistant strains (Figure 4). RL–chitosan nanoparticles demonstrated significant antimicrobial activity against *S. aureus*, with a minimal inhibitory concentration of 130 μg/mL [181].

#### 4.1.2. Polymeric Nanoparticles

**Figure 5:**
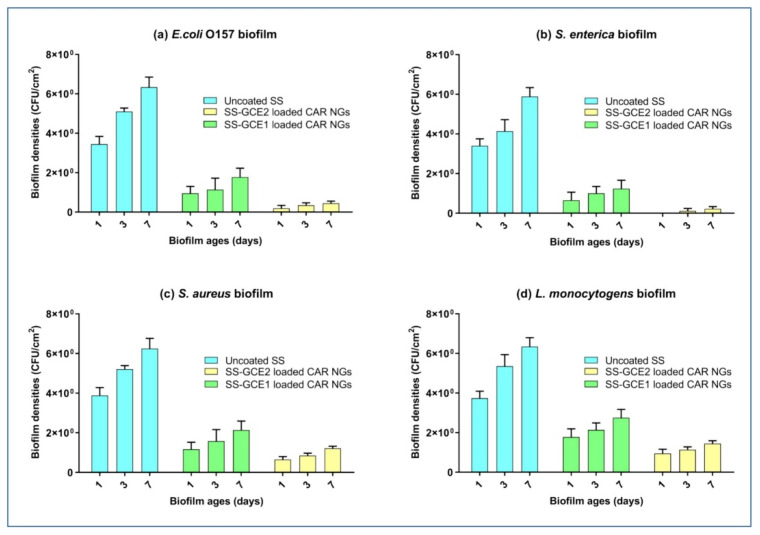
Antibiofilm inhibition role of the smart nanogel carrageenan nanogels (CAR NGs) based on carrageenan and green coffee extract against (**a**) *E. coli*, (**b**) *S. enterica*, (**c**) *S. aureus*, and (**d**) *L. monocytogenes* on the surface of the stainless steel. Reprinted with permission from [192], Copyright © 2024 Elsevier Ltd.

Polymeric nanoparticles are composed of biodegradable and biocompatible polymers, making them suitable for the controlled and regulated release of bioactive compounds, particularly those found in medicinal plants [182,183,184]. The efficiency of medicinal medications can be enhanced by engineering these nanoparticles with specific sizes, surface properties, and release patterns to ensure that the proper amount is delivered at the right time. To optimize the release kinetics, one must modify the polymer’s content or structure. Surface modifications with ligands or antibodies can be used to target specific cells or tissues, decreasing off-target effects and maximizing therapeutic potential [185,186]. Polymeric nanoparticles are an effective and versatile method for pharmaceutical delivery, particularly for treating localized infections or chronic conditions. Some polymeric nanoparticles, such as chitosan-based and poly (lactic-co-glycolic acid) (PLGA) nanoparticles, effectively break down bacterial biofilms [187]. Polymeric micelles and polymersomes are also utilized to deliver antivirulence drugs, such as bacterial toxin blockers or QS inhibitors, to lower pathogenicity [188,189]. Furthermore, poly-N-vinyl-caprolactam (PNVCL) nanoparticles can modify virulence factor expression, lowering bacterial pathogenicity [190]. These techniques have a high potential for treating chronic disorders caused by biofilms and those that are resistant to many medicines. Nanogels, particularly those based on carrageenan, show promise as antifouling and antibacterial coatings for various applications, including food packaging [191]. These smart nanohydrogels can be designed for the controlled release of preservatives and antimicrobial compounds. The antibiofilm inhibition role of the smart nanogel carrageenan nanogels (CAR NGs) based on carrageenan and green coffee extract exhibited potential antibiofilm activity against *E. coli*, *S. enterica*, *S. aureus*, and *L. monocytogenes* (Figure 5) [192].

#### 4.1.3. Metal Nanoparticles

**Figure 6:**
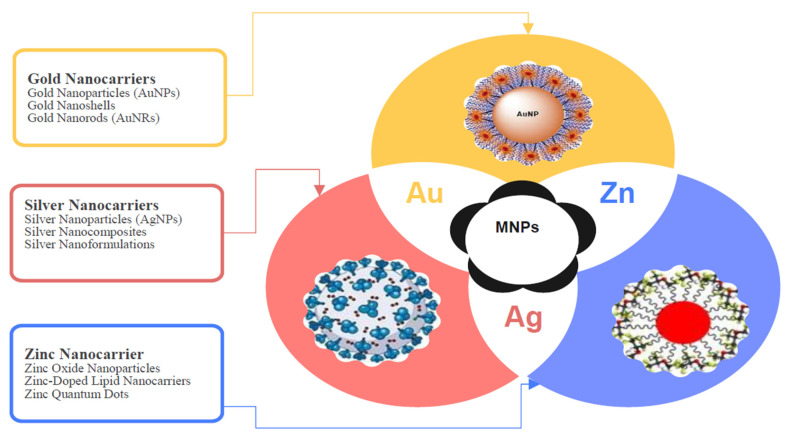
Metallic nanoparticles (MNPs) have antibiofilm and antivirulence properties.

Metal nanoparticles are becoming more and more popular in drug delivery systems because of their inherent antibacterial qualities, particularly those derived from gold and silver (Figure 6) [193,194,195]. By facilitating the efficient adsorption of bioactive chemicals and enhancing their contact with microbial cells, their high surface area-to-volume ratio can improve the therapeutic benefits of substances obtained from medicinal plants. Apart from their antibacterial properties, these nanoparticles can also serve as drug carriers, enhancing the stability and bioavailability of medications [196,197]. Metal nanoparticles (MNPs) including silver, gold, and zinc oxide inhibit microbial adhesion, QS, and biofilm development, resulting in significant antibiofilm and antivirulence properties [198,199]. Because of their small size, they can penetrate biofilms and produce ROS, which damage microbial cells. MNPs inhibit virulence factors such as toxins and proteases by interfering with essential proteins and enzymes [200,201,202]. Furthermore, they disrupt bacterial membranes, resulting in leakage and cell death. Because of these qualities, MNPs hold great promise in the fight against antibiotic-resistant illnesses [203]. At greater quantities, metal nanoparticles can be detrimental to human cells, which is why their use raises questions about cytotoxicity [204]. Thus, it is critical to carefully optimize their size, shape, and surface coating in order to strike a balance between therapeutic efficacy and safety while ensuring that the benefits outweigh the risks.

The exploration of novel therapeutic approaches has been prompted by the emergence of chronic biofilm-associated infections and antibiotic resistance. Nanoparticles provide flexible frameworks to counteract pathogenic threats through a variety of ways and have become a game-changing option [205]. These nanoscale agents are produced via scalable processes such as chemical reduction, biological synthesis, and hybrid approaches [206]. They are made from metallic, organic, and composite materials. Their design places a strong emphasis on balancing environmental and biocompatibility factors and efficacy. NPs facilitate the therapeutic penetration of the rigid biofilm through targeting EPS as well as facilitating the medication delivery [207]. Their action mechanisms include Gram-positive and Gram-negative bacteria, fungi, and other drug-resistant organisms. Innovations such as light-responsive activation and pH regulation enable more precise, spatiotemporal control over microbial eradication [208]. Moreover, NPs reduce microbial viability in a variety of ways, including physical damage to cell structures, the production of ROS, and interference with bacterial communication mechanisms such as QS. Despite NPs’ enormous promise, there are still challenges in enhancing their biocompatibility, scalable manufacture, and targeted dispersion [209].

Various parts of medicinal plants, such as leaves, roots, bark, flowers, and fruits, contain phytochemicals that act as natural reducing and stabilizing agents in the green synthesis of NPs. This sustainable method has been widely used to generate metallic NPs, including silver, gold, copper, and zinc oxide, with antimicrobial and antibiofilm properties. Table 2 summarizes recent developments in the use of medicinal plants for the green synthesis of NPs with antibiofilm and antivirulence properties. It outlines the types of NPs synthesized; the specific plant species and parts used; their biofilm inhibitory concentrations, underlying mechanisms of action; and the target microbial pathogens.

## 5. Synergy of Plant Compounds with Nanocarriers

The combination of plant-derived chemicals and nanocarriers is a novel strategy for fighting microbial diseases. The ability of chemicals derived from medicinal plants to reduce microbial pathogenicity and biofilm growth has received a lot of interest, especially when synthesized as nanoparticles [250,251,252]. Nanoformulations improve the bioavailability, stability, and targeted dispersion of bioactive compounds, making them more effective against treatment-resistant illnesses. This nanoencapsulation permits a controlled release at the infection site in addition to shielding the active components from early deterioration. Numerous plant-based compounds, such as berberine, curcumin, eugenol, and quercetin, have been successfully introduced as nanocarriers to metal nanoparticles, polymeric nanoparticles, and liposomes to improve their antimicrobial activity [253,254]. These nanoformulations target important microbiological processes such as QS suppression, EPS disruption, and biofilm adhesion and maturation [255,256,257]. In Gram-negative bacteria, the synergistic effect can inhibit receptor-mediated signaling pathways by sequestering or degrading QS molecules such as N-acyl homoserine lactones [258,259]. As a result, this disruption hinders the activation of virulence factors and biofilm-associated gene expression. Nanocarriers also disrupt the polymerization process, which is required to maintain the structural integrity of biofilms, by targeting enzymes involved in the production of extracellular polymeric compounds [260,261]. Because of this interference, the biofilm matrix is unable to adhere, mature, or consolidate, weakening the bacteria’s defenses and making them more susceptible to antimicrobial treatments.

## 6. Exploring the Potential and Pitfalls of Medicinal Plant Compounds

Medicinal plants have long been a source of bioactive compounds with antibiofilm and antivirulence characteristics that could potentially have therapeutic properties. On the contrary, the variety of phytochemical profiles within the medicinal plants poses a significant barrier to broad utilization. A variety of factors, including cultivation methods, extraction processes, ambient conditions, and geographic origin, contribute to differences in the function and chemical constituents of the plant compounds [262,263]. This disparity makes it more difficult to standardize formulations and obtain consistent results. Their therapeutic usage is further complicated by concerns about the stability, bioavailability, and safety of plant-derived compounds [264,265]. To fully utilize medicinal plants for therapeutic applications, these challenges must be addressed through sophisticated formulation techniques, rigorous safety investigations, and regulatory coordination.

### 6.1. Changes in Phytochemical Profiles

The variation in the phytochemical profiles of medicinal plant-derived substances is a significant barrier to their use for antivirulence and antibiofilm activities. The chemical compounds found in plants, also called phytochemicals, fluctuate significantly depending upon a number of variables, such as the plant’s origin, environment, and growth methods [266,267]. For instance, light exposure significantly alters the accumulation of scutellarin in *Erigeron breviscapus*, while the concentration of trans-cinnamaldehyde in *Cinnamomum cassia* varies non-linearly with plant age, peaking in mature bark but declining thereafter [268]. Similarly, in *Scutellaria baicalensis*, although the total flavonoid content remains stable, individual compounds such as baicalin fluctuate depending on the growth stage, with maximum levels observed just before full bloom [269].

Environmental stressors such as drought, salinity, and temperature extremes also influence secondary metabolite biosynthesis. In *Artemisia annua*, for example, stress can alter the production of artemisinin [270], while in *Glycyrrhiza glabra*, glycyrrhizin levels are similarly affected by abiotic conditions [271]. Moreover, even within the same cultivation area, as observed in *Panax notoginseng*, microenvironmental variation leads to inconsistencies in saponin profiles, impacting therapeutic reproducibility [272].

Furthermore, the concentration and composition of these phytochemicals can be further influenced by the extraction techniques used to separate bioactive substances [273,274,275]. The consistency and reproducibility of experimental results are affected by this variation, resulting in efforts to standardize formulations for therapeutic use being challenging.

To overcome these challenges, researchers must carefully choose and describe plant extracts. Standardizing plant extracts needs strict quality control procedures, such as determining the concentration of bioactive compounds, identifying chemical profiles using sophisticated methods (such as chromatography and spectrometry), and confirming the extracts’ stability under various storage conditions [276,277]. In clinical settings, this assures uniformity, reliability, and repeatability, ensuring that treatment outcomes remain uniform across trials and implementations. All of these features enhance the therapeutic potential of medicinal plant constituents in antiviral and antibiofilm therapy.

### 6.2. Concerns with Stability and Bioavailability

Many secondary metabolites found in medicinal plants are unstable and poorly bioavailable due to their susceptibility to physiological degradation [278,279,280]. This issue becomes especially problematic when attempting to transport these compounds efficiently to target sites within the body. Several factors contribute to reduced therapeutic efficacy, including enzymatic degradation in the gastrointestinal tract, rapid hepatic metabolism, and poor water solubility. For instance, curcumin, a polyphenol from *Curcuma longa*, exhibits potent therapeutic properties but demonstrates extremely low oral bioavailability, with up to 75% being excreted unchanged, with only trace amounts detected in systemic circulation. Even intravenous administration results in rapid biliary excretion, severely limiting its clinical utility. Other compounds, such as berberine and quercetin, similarly suffer from instability in aqueous environments and rapid metabolic clearance, which hinders their consistent therapeutic performance [281]. Nanoformulations provide an effective strategy to address these limitations. Through encapsulating plant-derived bioactives in nanoscale carriers, such as nanoparticles, nanoemulsions, or liposomes, researchers have achieved improvements in solubility, stability, and targeted release profiles [282,283,284]. For example, nanoemulsified curcumin has demonstrated significantly enhanced systemic retention and therapeutic efficacy compared to its crude form [281]. However, the development of these delivery systems brings new challenges. Optimizing the interaction between phytochemicals and nanocarriers requires careful physicochemical characterization, and understanding the altered pharmacokinetics of nanoencapsulated compounds remains a critical research priority. Future efforts should focus on refining nanoformulation platforms to minimize potential cytotoxicity while maximizing the bioavailability and therapeutic impact of medicinal plant-derived compounds.

### 6.3. Profiles of Safety and Toxicology

Since therapeutic plant components come from natural sources, they are frequently assumed to be inherently safe. However, this assumption should not take precedence over the necessity for comprehensive toxicological evaluation [285,286,287]. Extensive studies are required to assess both efficacy and potential adverse effects, especially when plant-derived compounds are used at high concentrations or in combination with other therapeutic agents [288]. Despite their promising medicinal potential, several phytochemicals have demonstrated organ-specific toxicity in preclinical studies. For instance, β-asarone, a neuroactive compound from *Acorus calamus*, has shown hepatotoxic effects in rodent models at higher doses [289], while vincristine, an alkaloid from *Catharanthus roseus*, is known to induce nephrotoxicity, neurotoxicity, and infertility [290]. Similarly, oleandrin from *Nerium oleander* has been associated with cardiotoxicity and renal impairment even at moderate doses, and tomentogenin, a pregnane glycoside from *Caralluma dalzielii*, caused hepatocellular distortion in vivo [291,292,293]. Other widely used botanicals, such as *Ginkgo biloba*, contain ginkgotoxin, which has been linked to neurotoxicity and potential hepatocarcinogenic effects [294]. Likewise, safranal from *Crocus sativus* has been shown to induce nephrotoxicity and metabolic disturbances in animal models [295].

Therefore, it is critical to conduct thorough preliminary studies on the cytotoxicity, immunogenicity and long-term safety of these compounds. Toxicological assessments must extend beyond the isolated phytochemicals to include the complete formulation, especially in nano-enabled delivery systems, and evaluate interactions with human biological systems [296,297]. A comprehensive understanding of their risk–benefit profile, including organ-specific toxicity, allergic potential, and genotoxic effects, is essential. Only after such rigorous safety validation can medicinal plant compounds be confidently employed in treating biofilm-associated infections or mitigating microbial virulence in clinical settings.

### 6.4. Challenges in Regulation and Commercialization

Significant scientific and regulatory constraints prevent the commercialization of bioactive chemicals derived from medicinal plants for therapeutic purposes [264,287,298]. The process of turning laboratory-based discoveries into clinically approved treatments is complex and resource-intensive, requiring thorough validation at several stages. These includes comprehensive preclinical and clinical research to support pharmacological activity and safety profiles, the standardization of plant extracts to guarantee batch-to-batch consistency, and the optimization of nanoformulation methods to improve bioavailability and therapeutic efficacy [299,300,301]. Before authorizing a product for sale, regulatory bodies like the European Medicines Agency (EMA) and the U.S. Food and Drug Administration (FDA) require a great deal of empirical data, including toxicological and pharmacokinetic evaluations [302]. These strict regulations add to the length of time and high expenses involved in the clinical translation of medicines produced from plants.

In addition to regulatory obstacles, the commercialization of bioactives derived from medicinal plants is further complicated by intellectual property (IP) issues [303,304,305]. The origin of many of these drugs can be found in the traditional therapeutic knowledge that is commonly found in indigenous societies. Concerns with equitable ownership and fair pay are brought up by the complicated legal and ethical issues regarding bioprospecting, benefit-sharing plans, and patentability. Frameworks like the Nagoya Protocol on Access and Benefit-Sharing are being implemented in an effort to address these problems [306], but there are still difficulties in coordinating global laws and guaranteeing adherence.

To successfully negotiate these commercialization and regulatory obstacles, a multidisciplinary strategy is essential. Regulatory scientists, pharmacologists, nanotechnologists, ethnobotanists, and lawmakers working together can help design standardized processes for the extraction, formulation, and validation of compounds made from medicinal plants [307,308]. Through this partnership, conventional knowledge and contemporary scientific discoveries are effectively integrated. Additionally, using state-of-the-art biotechnological developments like synthetic biology and CRISPR-based genetic changes may improve the scalability and repeatability of the manufacture of bioactive compounds [309,310]. It will be easier to commercialize compounds derived from medicinal plants for antivirulence and antibiofilm applications if regulatory and intellectual property issues are addressed through scientific and policy-driven interventions. This will make it easier to get into the market and utilize these bioactive compounds more widely.

## 7. Future Prospects and Emerging Directions

The future of antimicrobial medicine is being shaped by significant advances in high-throughput screening (HTS) and omics technologies, which provide exclusive prospects for identifying and characterizing bioactive compounds in medicinal plants [311,312,313]. HTS is an effective technique that enables researchers to instantly test thousands of compounds for antimicrobial properties utilizing automated equipment and modern analytical tools. These technologies allow for the quick identification of plant-derived compounds and provide insights into their molecular mechanisms, particularly for battling microbial biofilms and related pathogenicity [314,315]. The combination of customized medicine, along with targeted medicines, promises to increase the efficacy of plant-based treatments. Overall, interdisciplinary teamwork and translational research are essential to moving these findings from the laboratory to clinical settings and opening the door for new treatments for infections that are resistant to drugs [316,317,318]. The following section of this review covers the integration of modern advancements into the potential usage of medicinal plant components for a variety of therapeutic purposes.

### 7.1. Advances in Omics and High-Throughput Screening Technologies

HTS and omics technologies, such as proteomics, transcriptomics, metabolomics, and genomics, are transforming the search for compounds derived from medicinal plants [311,319,320]. These developments provide more effective characterization, which speeds up the production of new therapeutic medications. Large compound libraries may be quickly screened via HTS, making it possible to identify bioactive compounds from plants that might otherwise go undetected [321,322]. By evaluating gene expression, protein interactions, and metabolic pathways, omics technologies provide a better understanding of the molecular mechanisms behind the antivirulence and antibiofilm properties of plant substances.

Systems biology techniques can be used with multi-omics technologies to provide a comprehensive approach. This integration aids in clarifying the molecular processes that underlie interactions between phytochemicals and microbes. Using mass spectrometry and HTS, scientists may map protein–protein interactions, metabolic fluxes impacted by bioactive plant compounds, and global gene expression profiles [323,324]. This systems-level study contributes to the discovery of significant microbial pathways and regulatory networks that are influenced by phytochemicals, such as those implicated in QS, biofilm formation, and virulence expression.

In addition to improving prediction capacities, sophisticated computational modeling and machine learning algorithms make it easier to identify new drug targets and resistance mechanisms [325,326,327]. Synergistic phytochemical combinations can be logically designed to improve antimicrobial activity using network-based pharmacology techniques. Moreover, the identification of conserved microbial targets across a variety of pathogenic species is made possible by comparative multi-omics investigations, which raises the possibility for translation of phytochemical-based therapies. In addition to speeding up the screening of antimicrobial medicines derived from plants, this comprehensive method helps guide precision medicine tactics for fighting infections that are resistant to many drugs [328,329]. In the end, combining these state-of-the-art techniques transforms the creation of next-generation antimicrobials based on phytochemicals with increased potency, specificity, and sustainability.

### 7.2. Targeted Therapies and Personalized Medicine

The advent of customized medicine opens up new avenues for the focused therapeutic use of compounds produced from medicinal plants. In targeted therapies, individual patient profiles, including genetic composition, microbiota composition, and the particular pathogen causing the infection, may allow for the customization of treatments to optimize effectiveness and minimize negative effects [330,331]. Personalized nanoformulation approaches enhance therapeutic efficacy by tailoring nanoparticle characteristics to a patient’s physiological needs and increasing drug delivery [332,333,334]. These formulations reduce toxicity and off-target effects while boosting the bioavailability, stability, and targeted release of plant-derived compounds. Advanced research improves therapy outcomes by combining biomarker-driven approaches to identify treatment responses unique to each patient.

Using nano-delivery methods, combination treatments can co-administer many bioactive drugs to fight microbial resistance in a synergistic manner [335,336]. In order to combat multidrug-resistant microbes, these tactics are essential since they improve drug penetration and retention at infection sites. Overall, personalized nanoformulations have the potential to provide safer and more efficient antimicrobial medications.

## 8. Conclusions

The growing threat of biofilm-related problems and antibiotic resistance highlights the crucial need for new therapeutic techniques. Secondary metabolites derived from medicinal plants provide a promising solution to these problems. Some of these bioactive compounds, such as flavonoids, alkaloids, tannins, and essential oils, have potent antivirulence and antibiofilm properties. They reduce pathogenicity, degrade the extracellular polymeric matrix, and interfere with quorum sensing pathways. Nanoformulations, such as metal nanoparticles, polymeric nanoparticles, and lipid-based carriers, facilitate the target compounds stability and bioavailabilities. In addition, the targeted administration of phytochemicals is important for minimizing their inherent disadvantages, such as low solubility and quick disintegration. Synergistic combinations with conventional antibiotics improve their efficacy against infections that are resistant to several treatments. However, concerns about safety, phytochemical variety, and regulatory complexity require interdisciplinary collaboration as well as using front-line technologies such as high-throughput screening, multi-omics, and competitional modellings coupled with standardized clinical translation protocols. The future of antimicrobial interventions could be revolutionized by combining traditional understanding with nanotechnology and customized medicine to create long-term and precision-based therapies for persistent infections.

## Figures and Tables

**Table 2:** Medicinal plant-bioinspired nanomaterials in controlling biofilm and virulence properties.

Name of Nanoparticles	Plants and Their Parts Used for NP Synthesis	Biofilm Inhibitory Concentration (µg/mL)	Mechanisms of Action	Target Microorganisms	References
Silver NPs (AgNPs)	*Mespilus germanica* extract (phytosynthesis)	1.95–100 (MIC values ranged from 6.25 to 100)	Disrupts bacterial membrane, produces reactive oxygen species (ROS), downregulates biofilm (*mrkA*) and quorum sensing (*luxS*) genes.	*Klebsiella pneumoniae*	[210]
	Leaf extracts of *Semecarpus anacardium*, *Glochidion lanceolarium*, and *Bridelia retusa*	10–100	Inhibits exopolysaccharide (EPS) production, interferes with bacterial adhesion, disrupts biofilm matrix formation.	*Pseudomonas aeruginosa*, *Escherichia coli*, *Staphylococcus aureus*	[164]
	*Piper betle* aqueous extract	3–10	Inhibits EPS production, reduces hydrophobicity, inhibits quorum sensing-regulated virulence factors (prodigiosin, protease), downregulates biofilm and motility genes (*fimA*, *fimC*, *flhD*, *bsmB*, *flhB*, *rsbA*).	*Serratia marcescens*, *Proteus mirabilis*	[211]
	*Carum copticum* aqueous extract	-	Inhibits quorum sensing-controlled virulence factors (violacein, pyocyanin, pyoverdin, protease, elastase, rhamnolipids, prodigiosin), disrupts swimming and swarming motility, inhibits EPS production and biofilm formation.	*Chromobacterium violaceum*, *P. aeruginosa*, *S. marcescens*	[212]
	*Zingiber officinale* (ginger) and *Cinnamomum cassia* (cinnamon)	Ginger: 15.6–62.5 Cinnamon: 156–1250	Inhibits bacterial adhesion, disrupts biofilm matrix (EPS inhibition), reduces adherent cells on catheter surfaces, with ginger AgNPs also damaging intermolecular forces in biofilms.	*Enterococcus faecalis* and *Enterococcus faecium*	[213]
Gold NPs (AuNPs)	Aqueous extract of *Crinum latifolium* leaves	6.25–50	Inhibits germ tube formation, suppresses biofilm matrix production, reduces secretion of hydrolytic enzymes (phospholipase, proteinase, esterase, lipase, hemolysin), disrupts cell wall and membrane integrity.	*Candida* spp.	[214]
	Aqueous stem extract of *Tinospora cordifolia*	1000–1800	Inhibits pyocyanin production, reduces swarming and swimming motility, inhibits biofilm formation, reduces EPS secretion.	*P. aeruginosa* PAO1	[215]
	Aqueous extract of *Capsicum annuum*	25–200	Inhibits quorum sensing-mediated virulence factors (pyocyanin, pyoverdin, protease, elastase, rhamnolipid), reduces swimming motility, inhibits EPS production, disrupts biofilm matrix.	*P. aeruginosa* PAO1, *Serratia marcescens* MTCC 97	[216]
Zinc oxide NPs (ZnO NPs)	Aqueous extract of *Origanum vulgare* leaves	2–8	Inhibits quorum sensing (violacein pigment), downregulates QS genes (*cviL*, *vioA*, *vioB*, *vioD*, *vioE*), inhibits EPS production, disrupts biofilm formation.	*Chromobacterium violaceum* ATCC12472	[217]
	Aqueous leaf extract of *Cassia siamea*	0.5–20	Inhibits quorum sensing (reduces pyocyanin, pyoverdine, exoprotease, elastase, rhamnolipid), impairs EPS production, inhibits swimming motility, reduces violacein production, disrupts biofilm matrix.	*P. aeruginosa* PAO1, *C. violaceum* MCC2290	[202]
	Aqueous root extract of *Plumbago zeylanica*	200–400	Inhibits EPS production, disrupts bacterial adhesion, alters biofilm architecture, and eradicates established biofilms.	*E. coli*, *P. aeruginosa*, *S. aureus*	[218]
Copper Oxide NPs (CuO NPs)	Aqueous leaf extract of *Moringa oleifera*	1000	Disrupts biofilm matrix, reduces EPS production, inhibits quorum sensing-regulated virulence factors, causes membrane damage and cell lysis.	*Klebsiella pneumoniae*, *S. aureus*, *Acinetobacter baumannii*	[219]
	Polyherbal drug (Septilin) containing plant extracts	1–2.5	Inhibits biofilm formation, disrupts cell morphology, reduces EPS production, induces membrane damage.	*P. aeruginosa*, *MRSA* (*S. aureus*), *C. albicans*, *E. coli*	[220]
Iron Oxide NPs (Fe₃O₄ NPs)	Ultrasound-assisted synthesis using aqueous leaf extract of *Artemisia haussknechtii*	12.5–50	Inhibits biofilm formation, reduces EPS secretion, disrupts biofilm architecture, decreases bacterial spreading ability.	*MRSA* (*S. aureus*), *E. coli*, *S. marcescens*	[221]
Chitosan NPs	Encapsulation of plant essential oils into chitosan nanoparticles	25–150	Inhibits EPS production, disrupts biofilm matrix, reduces metabolic activity, damages internal and external bacterial structures.	*A. baumannii*	[222]
	*Lavandula angustifolia* (lavender) leaf extract	10–1500	Disrupts EPS production, reduces biofilm biomass and metabolic activity, alters biofilm morphology, decreases viability of biofilm-embedded cells.	*P. aeruginosa*, *S. aureus*, *C. albicans*	[223]
Selenium NPs (SeNPs)	Orange peel waste extract	MIC: 62.5–250	Inhibits biofilm formation, reduces biofilm biomass, disrupts surface adhesion of biofilm-forming cells.	*P. aeruginosa* PAO1, *K. pneumoniae*, *E. coli*, *S. aureus*	[224]
Titanium Dioxide NPs (TiO₂ NPs)	Grape seed extract rich in proanthocyanidins	-	Enhances intracellular uptake, induces intracellular ROS generation, inhibits planktonic cell proliferation, disrupts biofilm formation and biofilm matrix penetration.	*P. aeruginosa*, *S. saprophyticus*	[225]
Magnesium Oxide NPs (MgO NPs)	Flower extract of *Rosa floribunda charisma*	7.81–31.25	Disrupts biofilm formation, reduces bacterial growth, generates oxidative stress, inhibits bacterial membrane integrity.	*S. epidermidis*, *Streptococcus pyogenes*, *P. aeruginosa*	[226]
Cerium Oxide NPs (CeO₂ NPs)	*Arctium lappa* (burdock root) aqueous extract, encapsulated in nano-chitosan via sol–gel method	10–18	Inhibits biofilm formation by disrupting EPS production, induces oxidative stress (ROS generation), disrupts bacterial membrane integrity, reduces bacterial viability.	*S. aureus*, *P. aeruginosa*	[227]
Graphene Oxide–silver NPs (GO-Ag NPs)	Floral extract of *Lagerstroemia speciosa* (Banaba flower)	47–94	Inhibits biofilm formation, reduces EPS production, induces ROS generation, disrupts cell membrane integrity, downregulates biofilm-associated genes (*vicR*, *spaP*, *comDE*).	*S. mutans*, *Enterobacter cloacae*	[228]
Limonene-loaded alginate/collagen nanoparticles (LAC)	Encapsulation of limonene into alginate/collagen nanoparticles	0.781–1.56	Reduces biofilm formation by inhibiting OmpA and Bap biofilm gene expression, disrupts biofilm matrix integrity, and enhances ROS generation.	*A. baumannii*	[229]
PLGA NPs	Encapsulation of ethanolic extract of propolis (EEP) into poly(lactic-co-glycolic acid) (PLGA) nanoparticles via oil-in-water emulsion solvent evaporation method	1.25–2.5	Reduces adhesion, hyphal germination, and biofilm formation; downregulates virulence genes (*ALS3*, *HWP1*).	*C. albicans*	[230]
Liposomal NPs	Encapsulation of aqueous extract of *Punica granatum* into phospholipid-based nanoliposomes	2–2048	Inhibits biofilm formation, suppresses glucosyltransferase (GTF) activity, and reduces glucan production.	*S. mutans*	[231]
Silica NPs (SiO₂ NPs)	Hot aqueous leaf extract of *Thuja orientalis*	-	Inhibits biofilm, disrupts bacterial adhesion, interaction with bacterial DNA, and membrane.	*S. aureus*, *E. coli*	[232]
Silica nanoparticles (SNPs) loaded with *Eucalyptus globulus* oil	Synthesis of silica nanoparticles using the sol–gel method; eucalyptus oil encapsulated into SNPs	50	Inhibits biofilm formation by disrupting biofilm matrix, enhances oil penetration into biofilm, yet no direct virulence gene or quorum sensing suppression has been studied.	*E. coli* ATCC 25922	[233]
Nickel Oxide NPs (NiO NPs)	*Eucalyptus globulus* leaf extract	0.8–1.6	Inhibits biofilm formation, increases cell membrane permeability, disrupts cell morphology, generates ROS, damages DNA and proteins.	*P. aeruginosa*, *E. coli*, *S. aureus*	[234]
Carbon Quantum Dots (CQDs)	CQDs are synthesized from resveratrol (a phenolic compound from fruits)	100	Inhibits chemotaxis, biofilm formation, elastase, pyocyanin, rhamnolipid production; disrupts the pqs quorum sensing system (downregulates virulence genes, reduces PQS signaling).	*P. aeruginosa* PAO1	[235]
Palladium NPs (PdNPs)	Aqueous extract of *Allium sativum* (garlic)	3.125–50	Disrupts bacterial membrane integrity, inhibits biofilm formation, enhances antibiotic efficacy, promotes wound healing in vivo.	*S. aureus*, *P. aeruginosa*	[236]
	*Padina boryana* extract	31.25–125	Induces ROS generation, disrupts membrane integrity, inhibits biofilm formation, reduces CFU counts.	*S. aureus*, *E. fergusonii*, *A. pittii*, *P. aeruginosa*, *A. enteropelogenes*, *Proteus mirabilis*	[237]
Platinum NPs (PtNPs)	Aqueous extract of *Desmostachya bipinnata*	-	Disrupts biofilm matrix, inhibits bacterial adhesion, reduces plaque formation, exerts strong antibacterial activity against Gram-positive and Gram-negative bacteria.	*S. aureus*, *E. coli*	[238]
Zirconium Oxide NPs (ZrO_2_ NPs)	Aqueous ginger (*Zingiber officinale*) extract	5000–50,000	Disrupts membrane integrity (leakage of proteins, DNA, sugars), induces ROS generation, inhibits biofilm formation and quorum quenching via interaction with biofilm-associated proteins.	*A. baumannii*	[239]
Ag-ZnO NPs	Aqueous leaf extract of *Elephantopus scaber*	0.125	Disrupts bacterial growth and inhibits biofilm formation; antioxidant properties also confirmed, with possible ROS-mediated mechanism inferred.	*S. aureus*, *Bacillus subtilis*	[240]
Aluminum Oxide NPs (Al₂O₃ NPs)	*Citrus aurantium* (bitter orange) peel extract	-	Antimicrobial action disrupts microbial growth; antioxidant and anti-proliferative activities noted.	*P. aeruginosa*, *S. aureus*, *S. epidermidis*, *Klebsiella pneumoniae*, *C. albicans*, *A. niger*	[241]
Cadmium Sulfide NPs (CdS NPs)	Green synthesis using *Coronopus didymus* ethanolic extract	30,000–50,000	Disrupts bacterial membranes; ROS generation leading to DNA, RNA, and protein synthesis inhibition; cell lysis through interaction with thiol groups.	*E. coli*, *K. pneumoniae*, *S. aureus*	[242]
Piper betel leaf extract-coated hydroxyapatite nanoparticles (PBL-HAp)	Using hydroxyapatite (HAp) derived from eggshells, coated with Piper betel leaf extract using the microwave conversion method	-	Inhibits bacterial growth, inhibits biofilm formation, disrupts biofilm matrix integrity.	*S. aureus*, *E. coli*, *V. harveyi*, *P. aeruginosa*	[243]
AgNPs combined with molybdenum disulfide (AgNPs/MoS₂ Nanocomposite)	Tea tree essential oil (*Melaleuca alternifolia*); combined with exfoliated molybdenum disulfide nanosheets (MoS₂)	-	Disrupts bacterial membranes, induces DNA leakage, generates ROS, inhibits biofilm formation (99%).	*S. aureus*, *E. coli*	[244]
Lanthanum Oxide NPs (La₂O₃ NPs)	Combustion synthesis using *Centella asiatica* and Tridax plant leaf powders	50,000–100,000	Antibacterial action via cell membrane damage, moderate antifungal activity, photocatalytic ROS generation.	*S. aureus*, *E. coli*, *C. albicans*, *A. fumigatus*	[245]
CuO-Se Bimetallic NPs	*Lagenaria siceraria* leaf extract	7.8–250	Disrupts membrane and ROS generation; inhibits pyocyanin, protease, and pyoverdine production; inhibits biofilm formation via quorum sensing interference.	*P. aeruginosa*	[246]
Ag-Se Bimetallic NPs	*Orobanche aegyptiaca* extract with guar gum stabilizer	1000	Disrupts bacterial and fungal membranes, causes leakage of cytoplasmic content, inhibits biofilm formation, enhances ROS-mediated killing under UV exposure.	*S. aureus*, *E. coli*, *P. aeruginosa*, *C. albicans*	[247]
Cu-Fe Bimetallic NPs	*Hibiscus rosa*-sinensis flower extract	100	Disrupts biofilm network structure, induces bacterial cell death, inhibits biofilm formation.	*S. mutans*	[248]
Co-Zn-Ni Trimetallic Oxide NPs	*Cicer arietinum* leaf extract	-	Inhibits biofilm formation and binds to DNA Gyrase and surface protein G, suggesting anti-replication and anti-adhesion virulence inhibition.	*S. aureus*	[249]

## Data Availability

Not applicable.

## Abbreviations

EGCG	Epigallocatechin-3-gallate
Β	Beta
QS	Quorum sensing
EPS	Extracellular polymeric substance
